# WNT5B drives osteosarcoma stemness, chemoresistance and metastasis

**DOI:** 10.1002/ctm2.1670

**Published:** 2024-04-30

**Authors:** Rachel S. Perkins, Glenn Murray, Sarocha Suthon, Lindsey Davis, Nicholson B. Perkins, Lily Fletcher, Amanda Bozzi, Saylor L. Schreiber, Jianjian Lin, Steven Laxton, Rahul R. Pillai, Alec J. Wright, Gustavo A. Miranda‐Carboni, Susan A. Krum

**Affiliations:** ^1^ Department of Orthopaedic Surgery and Biomedical Engineering University of Tennessee Health Science Center Memphis Tennessee USA; ^2^ Center for Cancer Research University of Tennessee Health Science Center Memphis Tennessee USA; ^3^ Department of Pathology University of Tennessee Health Science Center Memphis Tennessee USA; ^4^ Department of Pathology Regional One Hospital Memphis Tennessee USA; ^5^ College of Medicine University of Tennessee Health Science Center Memphis Tennessee USA; ^6^ Department of Medicine University of Tennessee Health Science Center Memphis Tennessee USA

**Keywords:** cancer stem cells, metastasis, osteosarcoma, WNT5B

## Abstract

**Background:**

Treatment for osteosarcoma, a paediatric bone cancer with no therapeutic advances in over three decades, is limited by a lack of targeted therapies. Osteosarcoma frequently metastasises to the lungs, and only 20% of patients survive 5 years after the diagnosis of metastatic disease. We found that WNT5B is the most abundant WNT expressed in osteosarcoma tumours and its expression correlates with metastasis, histologic subtype and reduced survival.

**Methods:**

Using tumor‐spheroids to model cancer stem‐like cells, we performed qPCR, immunoblotting, and immunofluorescence to monitor changes in gene and protein expression. Additionally, we measured sphere size, migration and forming efficiency to monitor phenotypic changes. Therefore, we characterised WNT5B's relevance to cancer stem‐like cells, metastasis, and chemoresistance and evaluated its potential as a therapeutic target.

**Results:**

In osteosarcoma cell lines and patient‐derived spheres, WNT5B is enriched in stem cells and induces the expression of the stemness gene *SOX2*. WNT5B promotes sphere size, sphere‐forming efficiency, and cell proliferation, migration, and chemoresistance to methotrexate (but not cisplatin or doxorubicin) in spheres formed from conventional cell lines and patient‐derived xenografts. In vivo, WNT5B increased osteosarcoma lung and liver metastasis and inhibited the glycosaminoglycan hyaluronic acid via upregulation of hyaluronidase 1 (*HYAL1*), leading to changes in the tumour microenvironment. Further, we identified that WNT5B mRNA and protein correlate with the receptor ROR1 in primary tumours. Targeting WNT5B through inhibition of WNT/ROR1 signalling with an antibody to ROR1 reduced stemness properties, including chemoresistance, sphere size and SOX2 expression.

**Conclusions:**

Together, these data define WNT5B's role in driving osteosarcoma cancer stem cell expansion and methotrexate resistance and provide evidence that the WNT5B pathway is a promising candidate for treating osteosarcoma patients.

**Key points:**

- WNT5B expression is high in osteosarcoma stem cells leading to increased stem cell proliferation and migration through SOX2.
- WNT5B expression in stem cells increases rates of osteosarcoma metastasis to the lungs and liver in vivo.
- The hyaluronic acid degradation enzyme HYAL1 is regulated by WNT5B in osteosarcoma contributing to metastasis.
- Inhibition of WNT5B with a ROR1 antibody decreases osteosarcoma stemness.

## Graphical abstract

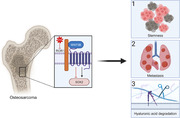

## 1 INTRODUCTION

Osteosarcoma (OS) is a solid tumour originating in the bone that primarily affects adolescents and children.^1^ Primary bone tumours in children make up 3%–5% of all childhood cancer diagnoses, and OS accounts for approximately 20%−40% of those bone cancers.^2^ Patients with primary disease have a 5‐year overall survival of 70% due to early surgical intervention and treatment with the cytotoxic MAP regimen of therapy [high‐dose methotrexate (M; MTX), doxorubicin (A; DOX), and cisplatin (P; CIS)].^1^, ^3^ MTX is an antifolate drug that works by inhibiting the folate pathway of DNA synthesis by specifically targeting dihydrofolate reductase (DHFR),^4^ while DOX and CIS cause DNA damage.^5^ Since OS has no targeted therapy and a high incidence of lung metastasis, survival rates for patients with metastatic disease are a dismal 20%.^3^ Moreover, OS patients frequently gain resistance to standard chemotherapy drugs, making chemoresistance a large contributing factor to failed OS treatment.^6^ Therefore, understanding the mechanisms of chemoresistance and metastasis is crucial to finding a successful treatment option for OS.

OS can gain resistance to general chemotherapy by several mechanisms, including increased drug efflux through membrane pumps (such as ABC (adenosine triphosphate (ATP)‐binding cassette) family members), alterations in DNA damage repair, and the effects of the tumour microenvironment, including the immune system.^7^ There are also specific mechanisms of resistance to different drugs. For example, resistance to MTX can be classified by an altered or increased affinity to DHFR and increases in multidrug resistance proteins (MRPs).^4^ Additionally, cancer stem cell (CSC) populations are a large contributor to chemoresistance in OS,^5^ and inhibition of the canonical WNT pathway in vivo has been shown to overcome the tumour's adaptive chemoresistance.^8^

The WNT family contains 19 genes and is divided into two main branches: β‐catenin‐dependent and β‐catenin‐independent (WNT/PCP and WNT/Ca^2+^) WNT signalling. β‐catenin‐dependent WNTs like WNT1, WNT3, WNT6, WNT7A and WNT10B regulate normal bone development.^9^ However, noncanonical β‐catenin‐independent WNTs, such as WNT5A and WNT5B, are less studied in bone.^10^

*WNT5B* is implicated in various human cancers, such as breast cancer, colorectal cancer and lung cancer.^10^ In these cancers, WNT5B has been shown to affect proliferation, differentiation, migration, invasion and tumour size.^10^ Although WNT5B has been shown to be overexpressed and correlated with poor overall prognosis in other cancer types, nothing is known about its role in OS.^10^ In fact, WNT5B is understudied in general, and its role in normal or diseased bone had not been explored prior to our work.^11^ We demonstrated that WNT5B regulates mesenchymal stem cells (MSCs) and inhibits osteoblast differentiation.^10^, ^11^ WNT5B has a high amino acid identity with WNT5A and is often assumed to have similar activities; however, WNT5B has opposite or unique functions compared to WNT5A.^10^ For instance, WNT5A promotes osteoblast differentiation, in contrast to WNT5B, which does not.

Due to WNT5B's inhibitory effect on osteoblast differentiation and positive regulation of MSCs, as well as its overexpression in various human cancers, we investigated the role of WNT5B in OS. In particular, we hypothesised that WNT5B plays a critical role in OS stem cells, thereby regulating stemness, chemoresistance, and metastasis. Therefore, this study determined the role of WNT5B in promoting the stem cell population of OS, increasing drug resistance to MTX, and increasing metastasis in vivo. Finally, we propose the therapeutic inhibition of WNT5B via a monoclonal antibody targeting its receptor ROR1.

## 2 METHODS

### 2.1 Reagents

Recombinant WNT5B and MTX were purchased from Bio‐Techne Corporation (Minneapolis, MN, USA). D10 antibody was provided by Oncternal Therapeutics, Inc. LGK‐974 (a pan‐WNT inhibitor that inhibits the secretion of all 19 WNT ligands) purchased from MedChemExpress (Monmouth Junction, NJ, USA). pX330‐U6‐Chimeric_BB‐CBh‐hSpCas9 was a gift from Feng Zhang (Addgene plasmid # 42230; http://n2t.net/addgene:42230; RRID:Addgene_42230).^12^

### 2.2 Adherent cells and spheres

All cells were maintained at 37°C and 5% CO_2_. Human OS cell lines 143B (RRID:CVCL_2270), MG63 (RRID:CVCL_0426), and SAOS‐2 (RRID:CVCL_0548) were purchased from ATCC. Cell lines were verified each year by short tandem repeat (STR) profiling and determined to be free of mycoplasma. 143B cells were grown in Dulbecco's Modified Eagle Medium (DMEM) with 10% fetal bovine serum (FBS), 1% penicillin‒streptomycin‒glutamine (PSG), Normocin, and 15 mg/mL 5′ BrdU. MG63 cells were grown in Minimum Essential Medium (MEM) with 10% FBS, 1% PSG, Normocin, nonessential amino acids, and sodium pyruvate. SAOS‐2 cells were grown in DMEM with 10% FBS, 1% PSG, and Normocin.

To select cells as spheres, cells were counted and plated on ultralow attachment Corning plates and grown in sphere media, which consisted of their respective media (143B and SAOS‐2 = DMEM and MG63 = MEM) without serum, and additional supplements of 4 µg/mL heparin (Sigma‒Aldrich), 1X B27 (Invitrogen) and 20 ng/mL bFGF (BD Biosciences). Spheres were cultured in a similar manner to what was previously described by our laboratory.^13^

### 2.3 Patient‐derived xenografts and patient‐derived xenograft‐derived spheres

Patient‐derived xenografts (PDX) were obtained from St. Jude Children's Research Hospital and expanded via subcutaneous injection in NSG mice before establishing cell lines and spheres. At PDX tumour collection, tissue was digested to obtain only tumour cells. At this point, cells were grown either in conditions to select for cell lines or spheres. All PDX cell lines were grown in DMEM with 10% FBS, 1% PSG, and Normocin. In order to select cells as spheres, freshly digested tumour cells were plated on ultralow attachment Corning plates in the sphere‐culture medium: DMEM, 1% PSG, Normocin, 4 µg/mL heparin (Sigma‒Aldrich), 1X B27 (Invitrogen) and 20 ng/mL bFGF (BD Biosciences).

### 2.4 IncuCyte

Cells were plated and treated with recombinant WNT5B, LGK‐974 or MTX on Corning 96‐well flat‐bottomed plates. The growth rate was assessed using an IncuCyte (Sartorius) to image cells every 2−4 h until cells reached confluence.

Cell migration was assessed by wound healing. Cells were plated and allowed to grow to confluence and subjected to a single scratch across the surface of the cell growth area using a wound maker (Sartorius). Cells were washed, placed in the IncuCyte, and imaged every 2 h for 24 h. The wound closure rate was analysed as a measure of migration in the adherent population of cells.

### 2.5 Lentiviral infection and knockout/knockdown experiments

Lentiviral infection was performed with a master mix of growth media, 8 µg/mL polybrene and 5 µL of viral plasmid (scrambled, *WNT5B*, or luciferase) in a 6‐well plate. Cells were centrifuged for 45 min at 1400×*g* and placed in the incubator. Twenty‐four hours after infection, the cells were washed and cultured with growth media supplemented with puromycin or zeocin. Scrambled virus: Shc002v (puromycin, Sigma), *WNT5B* virus: (TRCN0000437705, Sigma, puromycin) and Luciferase virus: (firefly) (LVP1223 EF1a‐Luciferase, GenTarget, zeocin).

CRISPR‐cas9 was performed using the Lipofectamine 3000 Reagent protocol (ThermoFisher). Three different guide RNA (gRNA) sequences were designed to target *WNT5B*: gRNA1: Forward, CACCGCCCGCGGCGCTCACCGCGT, Reverse, AAACACGCGGTGAGCGCCGCGGGC; gRNA2: Forward, CACCGCGGACAACGCATCTGTCTT, Reverse, AAACAAGACAGATGCGTTGTCCGC; gRNA3: Forward, CACCGTACGGCTACCGCTTCGCCA, Reverse, AAACTGGCGAAGCGGTAGCCGTAC. Each gRNA was cloned into an all‐in‐one pU6‐sgRNA‐CAS9‐P2A‐GFP plasmid, which was modified from pX330 (Addgene #42230).^12^ All plasmids were sequenced to confirm successful ligation. The CRISPR master mix (2500 ng of plasmid (empty vector or targeting *WNT5B*), 5 µL of P3000 Reagent, and 3.75 µL Lipofectamine) was incubated for 15 min. Cells were then treated with the CRISPR complex and allowed to transfect for 24 h before undergoing single‐cell clonal expansion to ensure 100% knockdown efficiency.

siRNA was also performed using the Lipofectamine 3000 Reagent protocol (ThermoFisher). Cells were treated with 100 nM of either a scrambled control or *ROR1* (Santa Cruz: sc‐76424) siRNA combined with Lipofectamine Reagent for 15 min and then added to the cells for transfection. Cells were cultured for several days and then selected as spheres for downstream applications.

### 2.6 RNA isolation and qPCR

As previously described,^11^ RNA was isolated by TRIzol reagent (Invitrogen), quantified by NanoDrop, and converted to cDNA at a concentration of 2000 ng using Maxima First Strand cDNA Synthesis Kit (ThermoFisher Scientific). The primer sequences used for qPCR are listed in Table S1. cDNA was quantified using SYBR Green Master Mix (ThermoFisher Scientific). The qPCR cycling conditions for SYBR Green were initiated at 95°C for 10 min, followed by 40 cycles of 95°C for 15 s and 60°C for 1 min, and then detection of the melt curve. β‐actin (*ACTB*) was used as the normalising gene for all data analyses.

### 2.7 Protein extraction and immunoblotting

Protein was isolated in EBC (50 mM Tris‐HCl, 120 mM NaCl, 0.5% NP‐40, pH 8.0) lysis buffer containing Complete Protease Inhibitor Cocktail (Roche) and PhosSTOP (Roche). Protein concentrations were quantified using Coomassie blue staining and a bovine serum albumin (BSA) standard. Protein (10−20 µg) containing dithiothreitol (DTT) and sodium dodecyl sulfate (SDS) loading buffer was loaded onto 10‐ or 15‐well 5%−12% SDS‐polyacrylamide gels and wet transferred onto a polyvinylidene difluoride membrane (Bio‐Rad). Nonspecific proteins on the membrane were blocked with either 5% milk, 5% BSA, or 5% milk with 1% BSA in TBST (Tris‐buffered saline, 0.1% Tween 20) at room temperature for 1 h. Then, the blots were probed with primary antibodies (listed in Table S2), which were left while rocking at 4°C overnight, followed by incubation with the secondary rabbit or mouse antibody conjugated to horseradish peroxide. The immunoblots were then exposed to Radiance Plus chemiluminescent substrate (Azure Biosystems) and imaged using iBright Imaging Systems (ThermoFisher Scientific) and β‐actin (ACTB) served as the loading control. Blots were quantified using NIH's ImageJ densitometric analysis, with normalization to ACTB.

### 2.8 Sphere size

Spheres were plated on day 0 and treated with varying doses of drugs (MTX, LGK, or D10) or recombinant proteins in triplicate on days 0 and 3 and imaged on day 5. Ninety‐six‐well plates were scanned using an EVOS FL Auto microscope, and four images per well were collected with a 10× objective, yielding 12 images per group. Images were then analysed for sphere number and sphere area using NIH's ImageJ software (RRID:SCR_003070) and a macro that was developed by Ivanov et al.^14^ and modified by our lab. Due to the nature of sphere debris and background, all images were subjected to a further round of analysis on ImageJ, checking every image for missing or incorrect sphere measurements. Raw data were run through GraphPad Prism's (RRID:SCR_002798) outlier test to screen out 0.1% of outliers within groups. Then, the data were graphed and analysed with statistical measures: ANOVA and *t*‐tests.

### 2.9 Sphere migration

143B control and *WNT5B* KO spheres were grown for 3 days in 96‐well low attachment plates, and then, using wide‐based pipet tips, spheres were diluted and plated onto grid plates to select for droplets with single spheres. These single spheres were subsequently plated onto collagen type 1 (Corning)‐coated 96‐well plates and treated with 10 ng/mL recombinant WNT5B or 20 µM LGK‐974. Spheres were allowed to settle to the collagen and were imaged at intervals of 0, 6, 12, and 24 h. The sphere migration area was measured using ImageJ.

### 2.10 Limiting dilution assays

143B Control and *WNT5B* KO cells were plated in decreasing densities and allowed to grow for 3 days. Then, all 96 wells were scanned at 4X, and spheres were counted using Photoshop's (RRID:SCR_014199) counting tool and plotted using GraphPad Prism software.

### 2.11 Animal experiments

All animal studies were approved by UTHSC's Institutional Animal Care and Use Committee. Cells tagged with luciferase (see lentivirus method above) to aid in tumour monitoring were implanted into male NSG mice (10–14 weeks old). Mice bearing tumours were monitored weekly for luciferase with an IVIS Lumina XRMS (PerkinElmer), as well as every other day weight and caliper measurements. Animal endpoints were determined by 15% weight loss or a maximum tumour measurement of 1.5 cm in any direction (maximum volume of 3.4 cm^3^). Tumour volume was calculated using a caliper to measure length × width × height.

Intratibial injections of tumour cells were performed under isoflurane gas with a volume of 20 µL consisting of 1:1 media:Matrigel (Corning) and 200,000 cells injected per mouse. At the endpoint, tumours and metastases were collected in TRIzol for RNA purification.

Orthotopic implantation of OS cells followed the method developed by Talbot et al. to remove a small piece of the tibial head and replace it with a collagen implant embedded with tumour cells.^15^ Spheres were cultured for 3 days and then dissociated using 2.5% trypsin and a FACS (fluorescence‐activated cell sorting) filter cap to ensure the presence of single cells. A total of 5,000 stem cells were resuspended in 15 µL of 1:1 Type I Collagen (Corning) in RPMI and drop‐plated in a 12‐well plate to make the implant. Collagen implants were allowed to solidify for 20−30 min at 37°C and 5% CO_2_ before sphere media was added. Collagen implants in media were given 24 h to set before implantation. The rest of the procedure was performed as described by Talbot et al.^15^

### 2.12 Immunohistochemistry

At humane endpoints, tibial and femoral bones with primary tumours and lungs/livers containing metastatic tumours were collected and decalcified (bones only) in DeCal Stat reagent (StatLab), formalin‐fixed, and embedded in paraffin. Samples were sent to the UTHSC Pathology Core for slide preparation. Human OS tissue microarrays were purchased from Biomax. Paraffin‐embedded samples were deparaffinised, and antigen retrieval was performed with citrate buffer that was heated to 100°C. Then, samples were added once the buffer reached 95°C and left to cool in the buffer until the temperature reached 55°C. Nonspecific antigens were blocked with 10% normal goat serum in PBS for 30 min at room temperature. Primary antibodies (Table S2) were added and incubated at 4°C overnight in a humidified chamber. Samples were washed with PBS, incubated with secondary antibodies and visualised by peroxidase/DAB solution (Dako) together with hematoxylin counterstaining. Tissue microarray cores were scored without bias by three independent people using a bone‐specific immune reactive scoring index of 0 (negative) to 12 (strongly positive).^16^

### 2.13 Quantification of metastases area and mitotic activity

Tissue samples were stained with hematoxylin and eosin. To measure metastasis area and metastatic burden, the lungs and liver were scanned using 4× image stitching on the EVOS FL Auto microscope. Then, NIH's ImageJ software was used to outline metastatic foci and measure area. For metastatic burden, the total area of metastasis was divided by the total area of the lung or liver, to give a percentage of healthy versus metastatic tissue. CaptaVision+ software (ACCU‐SCOPE, Commack, NY, USA) was used for digital manual class counting of the number of mitoses per millimetre squared. A total of 1,392 individual 0.035 mm^2^ digital microscopic fields were analysed among control and knockout (KO) tissues from bone, lung and liver. Counting of mitoses was performed in microscopic hot spot locations. Karyorrhexis and apoptosis/pyknosis were excluded from the mitotic count.

### 2.14 Alcian blue staining

Paraffin‐fixed slides were deparaffinised and placed in 1% alcian blue staining solution (pH 2.5) for 30 min. Then, slides were washed and counterstained in 0.1% nuclear fast red solution for 5 min. Slides were washed, dehydrated and mounted with a resinous mounting medium (Cytoseal). For alcian blue with hyaluronidase, slides were deparaffinised, then incubated in 1 unit/mL hyaluronidase from *Streptomyces hyalurolyticus* (Millipore) or PBS +0.01% BSA for 1 h at 37°C. Following incubation, slides were washed in running water for 10 min, and then stained with the alcian blue protocol above.

### 2.15 Immunofluorescence

Human OS cell lines were grown in either adherent or sphere conditions for 72 h. Adherent cells were grown in 8‐well chamber slides. Spheres were grown in non‐adherent well plates and collected in 1.5 mL Eppendorf tubes for staining.

Whole spheres were collected and then spun down at every stage to remove media, wash buffers, and antibodies. Cells were fixed with 4% paraformaldehyde and then washed with 1X PBS before blocking.

For paraffin embedding, spheres were collected and embedded in Histogel, paraffin‐embedded, and sectioned. Samples were deparaffinised, then antigen retrieval was performed as stated in the immunohistochemistry (IHC) method section.

Finally, both whole spheres and embedded slices were blocked with 0.2% Tween‐20, 1% normal goat serum, 1% BSA, and 0.02% sodium azide in 1X PBS. Specific antibodies (Table S2) were added overnight at 4°C. Secondary rabbit/mouse antibodies conjugated to a red or green fluorophore were incubated for 1 h at room temperature protected from light and then mounted with Vectashield Mounting Media with DAPI (4',6‐diamidino‐2‐phenylindole, H‐1200, Vector Laboratories) to stain the nuclei.

### 2.16 Statistics

All experiments represent both biological and experimental replicates. Statistical analyses, such as one‐way ANOVA, t‐tests, and linear regression plots, were performed using GraphPad Prism software.

### 2.17 Data availability

WNT expression in OS samples was obtained from NCI's TARGET database (available for download at https://ocg.cancer.gov/programs/target/using‐target‐data) and St. Jude Cancer Research Center's Pediatric Cancer Data Portal (https://pecan.stjude.cloud/). *WNT5B* expression in metastatic samples was obtained from NCBI GEO (GSE32981^17^). *WNT5B* expression correlated with survival, *ROR1*, and histological subtype was obtained from the R2 Genomics Analysis and Visualization Platform (http://r2.amc.nl) using the data of Kuijjer *et al*.^18^

## 3 RESULTS

### 3.1 WNT5B is overexpressed in osteosarcoma and correlates with metastasis and reduced survival outcomes in patients

**FIGURE 1:**
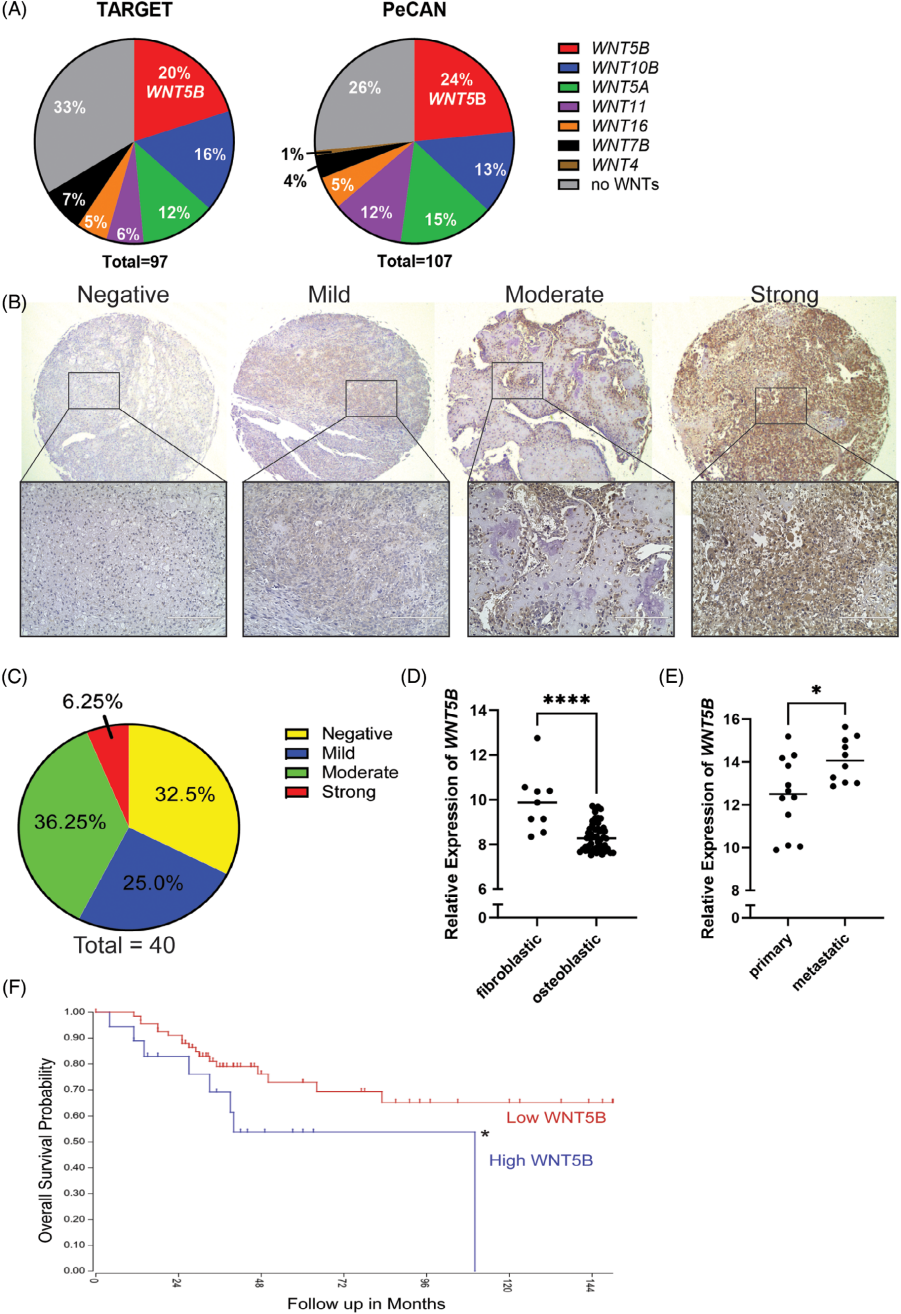
WNT5B is expressed in osteosarcoma patients and correlates with survival and metastasis. (A) Percentage of osteosarcoma (OS) patient samples expressing a WNT ligand. RNA sequencing data from the TARGET database, *n* = 97, and PeCan Data Portal, *n* = 107. (B) 4× and 20× images of WNT5B staining on osteosarcoma primary tumour tissue microarray (TMA), scale bar = 200 µm. *n* = 40. (C) Staining intensity scoring from the tissue microarray in (B). *n* = 40. (D) *WNT5B* expression analysis of osteosarcoma tumours sorted into subtypes, *n* = 62. ****p < .0001, data from Kuijjer et al.^18^ (E) *WNT5B* expression from global gene expression profiling of human osteosarcoma primary tumours vs. metastatic, *n* = 23. *p < .05, data from GSE32981.^17^ (F) Kaplan‒Meier survival curve of *WNT5B* in OS, data from R2: Genomics Analysis, Kuijjer et al.^18^ High *WNT5B* expression = blue line, low expression = red. Expression cutoff = 171.2; cutoff mode = scan. *n* = 86 total, *n* = 18 high, *n* = 68 low. *p < .05.

Using publicly available RNA sequencing datasets from NIH's TARGET database^19^ (dbGaP phs000468.v3.p1) and St. Jude Children's Research Hospital's Pediatric Cancer Data Portal (PeCan)^20^ we identified *WNT5B* as the most expressed WNT ligand across OS samples, comprising approximately 20%−24% of OS cases (Figure 1A). We validated the expression of WNT5B protein by performing IHC on a commercially available OS tissue microarray (TMA) consisting of primary tumours from 40 individuals (Figure 1B). The IHC samples were scored on a scale of 0−12 (based on intensity and percent positivity) and classified into negative, low, moderate, and strongly positive categories. WNT5B scored a moderate to strong expression in 42.5% of the patient samples (Figure 1B,C).

Subsequently, data from publicly available sources were analysed for correlations between *WNT5B* and histological subtype, metastasis, and survival. Expression array data from Kuijjer *et al*.^18^ indicate that *WNT5B* is more highly expressed in fibroblastic OS samples than in osteoblastic samples (Figure 1D). This was not surprising since our lab's prior work on normal bone concluded that WNT5B inhibits osteoblast differentiation and enhances MSC self‐renewal.^11^ Additionally, *WNT5B* expression was significantly higher in unmatched metastatic OS samples than in primary tumour samples^17^ (Figure 1E). Finally, high *WNT5B* expression correlated with a reduced overall survival time (Figure 1F). These results provide a foundation that WNT5B is critical in OS metastasis and survival.

### 3.2 WNT5B expression is increased in stem‐like populations of cells compared to adherent cell populations

Human OS cell lines were characterised to determine basal expression levels of WNT5B. MG63 and 143B cells had high and moderate mRNA and protein expression of WNT5B, respectively, while SAOS‐2 cells had low expression (Figure S1A–C). Subsequently, short hairpin (shRNA) and CRISPR *WNT5B* KO were generated and validated in the 143B cell line (Figure S1D–I). The results of proliferation and migration assays in these three adherent cell lines indicated that the addition of recombinant WNT5B (rWNT5B), inhibition of WNT signalling with LGK‐974 (to inhibit the secretion of all WNT ligands), and the KO/knockdown of *WNT5B* did not affect the proliferation rate or migration in scratch assays (Figure S2). These results, and the correlation between *WNT5B* and fibroblastic subtypes, which are less differentiated and more stem‐like, as well as the upregulation of *WNT5B* in metastatic samples, led us to hypothesise the involvement of the CSC subpopulation. OS cell lines and patient‐derived samples readily form spheres (also known as sarcospheres) by culturing methods that have been well‐established by our laboratory and others.^21^, ^22^ These spheres represent the CSC (or stem‐like) population of cells and show increased expression of the stemness markers *SOX2, OCT4*, and *NANOG* (Figure S3A). In addition, 143B, MG63, and SAOS‐2 spheres exhibit more chemoresistance to MTX than adherent cells (Figure S3B–D). Others have shown that cells grown as spheres have greater tumour growth in vivo.^23^ Together, these findings demonstrate the characteristics of CSCs.^6^

**FIGURE 2:**
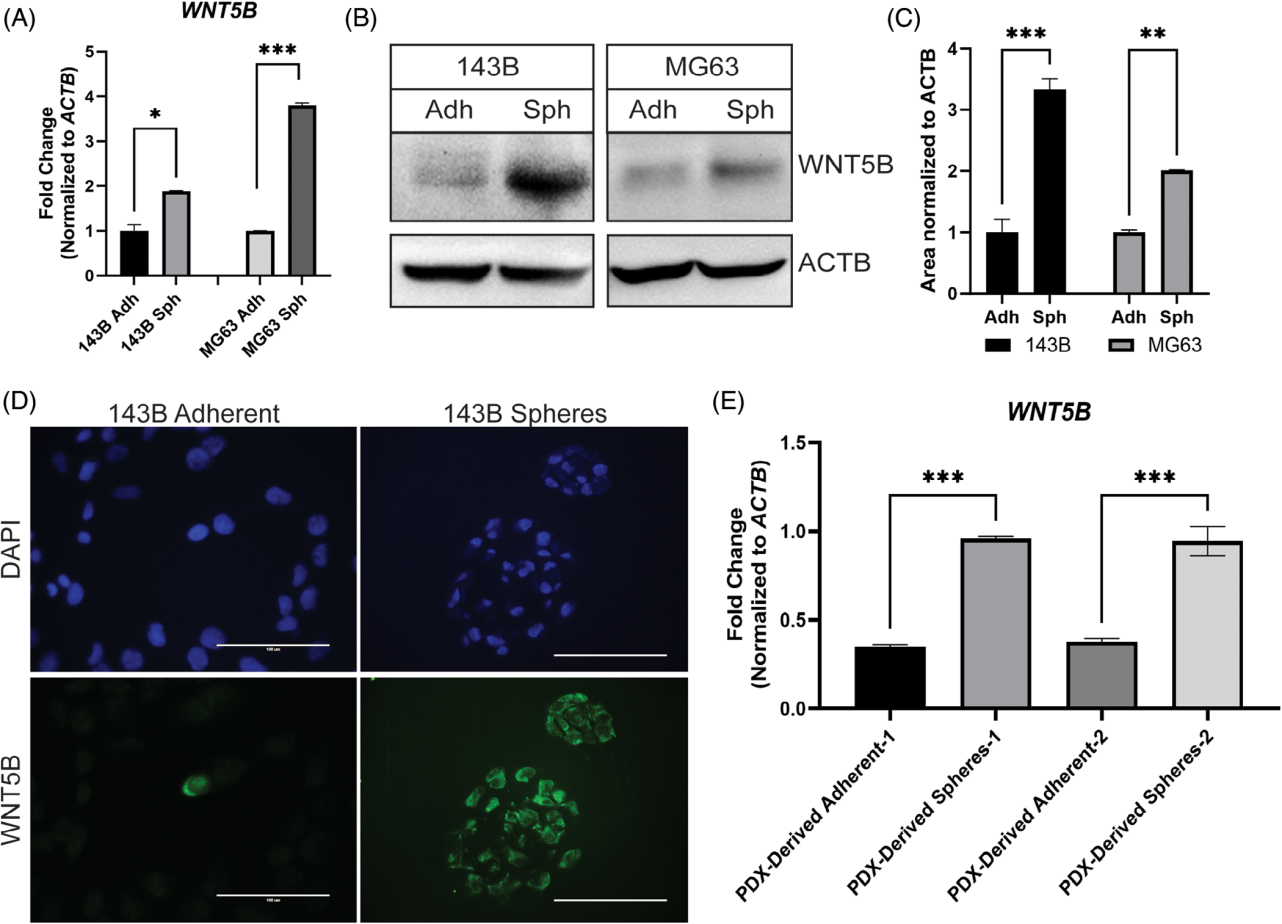
WNT5B expression is enhanced in osteosarcoma stem cells. (A) *WNT5B* expression in spheres (Sph) compared to adherent cells (Adh) by qPCR and normalised to *ACTB*, *n* = 3, *p < .05, ***p < .001. (B) WNT5B western blot in spheres (Sph) and adherent cells (Adh). Normalised to β‐Actin (ACTB), *n* = 3. (C) Quantification of western blots from (B), ***p < .001, **p < .01. (D) Immunofluorescence of 143B adherent cells and paraffin‐embedded spheres. Representative 40× microscopy images, WNT5B = green, DAPI nuclear stain = blue. Scale bar = 100 µm. (E) *WNT5B* expression analysis on RNA from two independent osteosarcoma PDX‐derived samples grown as adherent cell lines or spheres. qPCR normalised to *ACTB, n* = 3, ***p < .001.

MG63 and 143B adherent versus sphere populations were compared by both qPCR and western blot, and the CSCs had higher expression of WNT5B than the adherent populations (Figure 2A–C). We verified that this expression difference was not only because of the media by performing a qPCR comparing 143B adherent cells, 143B spheres, and 143B plated as spheres with adherent media added. There was no significant difference in *WNT5B* expression between the two sphere groups and both had significantly more expression of *WNT5B* than the adherent group (Figure S3E). Further validation of increased expression of WNT5B in spheres was performed using immunofluorescence (IF) of adherent and sphere populations, which demonstrated a major increase in the number of cells expressing WNT5B in CSCs compared to adherent cells (Figure 2D). The expression of *WNT5B* in two independent PDX OS samples grown as either adherent cells (PD cells) or spheres was assessed to confirm that the *WNT5B* expression was significantly higher in spheres than in adherent cells (Figure 2E).

### 3.3 WNT5B increases the size of osteosarcoma spheres

**FIGURE 3:**
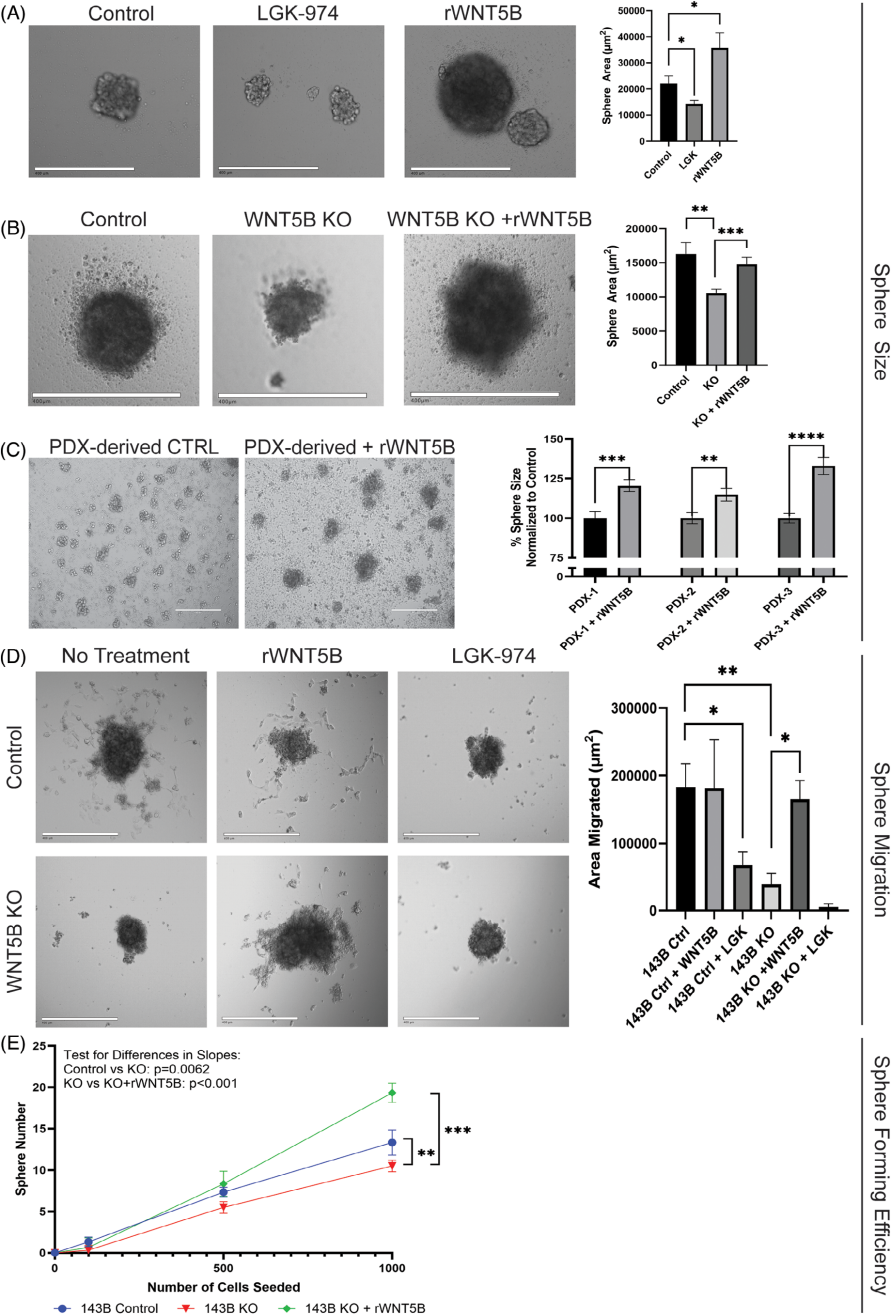
WNT5B drives osteosarcoma sphere size, sphere migration, and sphere‐forming efficiency. (A) 143B spheres treated with either 20 µM LGK‐974 or 10 ng/mL rWNT5B for 72 h. Sphere size quantified using ImageJ. *n* = > 40 spheres per group, *p < .05, Scale bar = 400 µm. (B) 143B Control and *WNT5B* KO spheres treated with 10 ng/mL rWNT5B for 72 h. Sphere area quantified using ImageJ, *n* = > 70 spheres per group, **p < .01, ***p < .001, scale bar = 400 µm. (C) Representative 10× microscopy images of osteosarcoma PDX‐derived spheres treated with 50 ng/mL rWNT5B compared to the untreated control, followed by ImageJ quantification of the sphere area. *n* = > 150 spheres per group, **p < .01, ***p < .001, ****p < .0001, scale bar = 400 µm. (D) 143B control and *WNT5B* KO spheres plated as single spheres in collagen‐coated wells and treated with either 10 ng/mL rWNT5B or 20 µM LGK‐974. Spheres were left to migrate onto the collagen for 24 h. Spheres were imaged at 10×, and the area of migration was quantified using ImageJ. *n* = average of 7 wells/group, *p < .05, **p < .01, scale bar = 400 µm. (E) 143B control (blue), *WNT5B* KO (red), and *WNT5B* KO + 10 ng/mL rWNT5B (green) cells were plated in sphere‐forming conditions in a limiting dilution and allowed to form spheres. Whole wells were scanned at 4×, and the sphere number was counted using Adobe Photoshop. Experiments were repeated three times with similar results, **p < .01, ***p < .001. KO, Knockout.

Since WNT5B expression is higher in CSCs than in adherent cells, the role of WNT5B in the CSC population was further investigated. The addition of rWNT5B significantly increased the size of 143B OS spheres, and the inhibition of WNT5B through LGK‐974 significantly reduced sphere size (Figure 3A). Similarly, when *WNT5B* is KO from 143B cells, sphere size is significantly reduced, which can be rescued with the addition of rWNT5B (Figure 3B). Additionally, spheres from three different PDX tumours were treated with rWNT5B to show the same effect: all three samples grew significantly larger with rWNT5B treatment (Figure 3C). This phenotype was also verified in other OS cell lines. WNT5B increased the sphere size of SAOS‐2 cells, which have low expression of endogenous WNT5B. WNT5B did not further enhance the sphere size in MG63 cells, which already highly express WNT5B (Figure S3F,G). MG63 and SAOS‐2 cells both showed a significant decrease in sphere size after treatment with LGK‐974 (Figure S3F,G).

### 3.4 WNT5B enhances the migration and sphere‐forming efficiency of osteosarcoma cancer stem cells

To assess WNT5B's impact on the migratory potential of our CSCs, collagen migration assays were performed, wherein single spheres were plated on collagen‐coated plates and allowed to migrate for 24 h (Figure 3D). The area of migration was quantified using ImageJ. 143B CSCs maintained the ability to migrate out from the original sphere, while 143B *WNT5B* KO spheres were unable to migrate. This phenotype was rescued with the addition of rWNT5B and inhibited with the addition of LGK‐974.

Sphere‐forming efficiency is a measure of stemness in CSCs.^24^ 143B *WNT5B* KO CSCs contained less sphere‐forming ability than 143B control cells, and this sphere‐forming efficiency was rescued with the addition of rWNT5B and inhibited with LGK‐974 treatment (Figure 3E). This demonstrates a difference in stem cell number between 143B control and 143B *WNT5B* KO cells and that rWNT5B rescues the *WNT5B* KO phenotype.

### 3.5 WNT5B drives expression of the stemness gene SOX2

Given the phenotypic role of WNT5B in the OS CSC population, we hypothesised that WNT5B might affect the expression of stemness markers. The three main stemness transcription factors (*SOX2, NANOG*, and *OCT4*)^6^ were all upregulated in the sphere versus the adherent population (Figure S3A); however, only *SOX2* significantly changed in response to *WNT5B* KO (Figure S3H). We performed a time course experiment with rWNT5B and determined that the highest expression for *SOX2* mRNA was at 6 h compared to either 3 h or 24 h in the 143B control spheres (Figure S3I).

**FIGURE 4:**
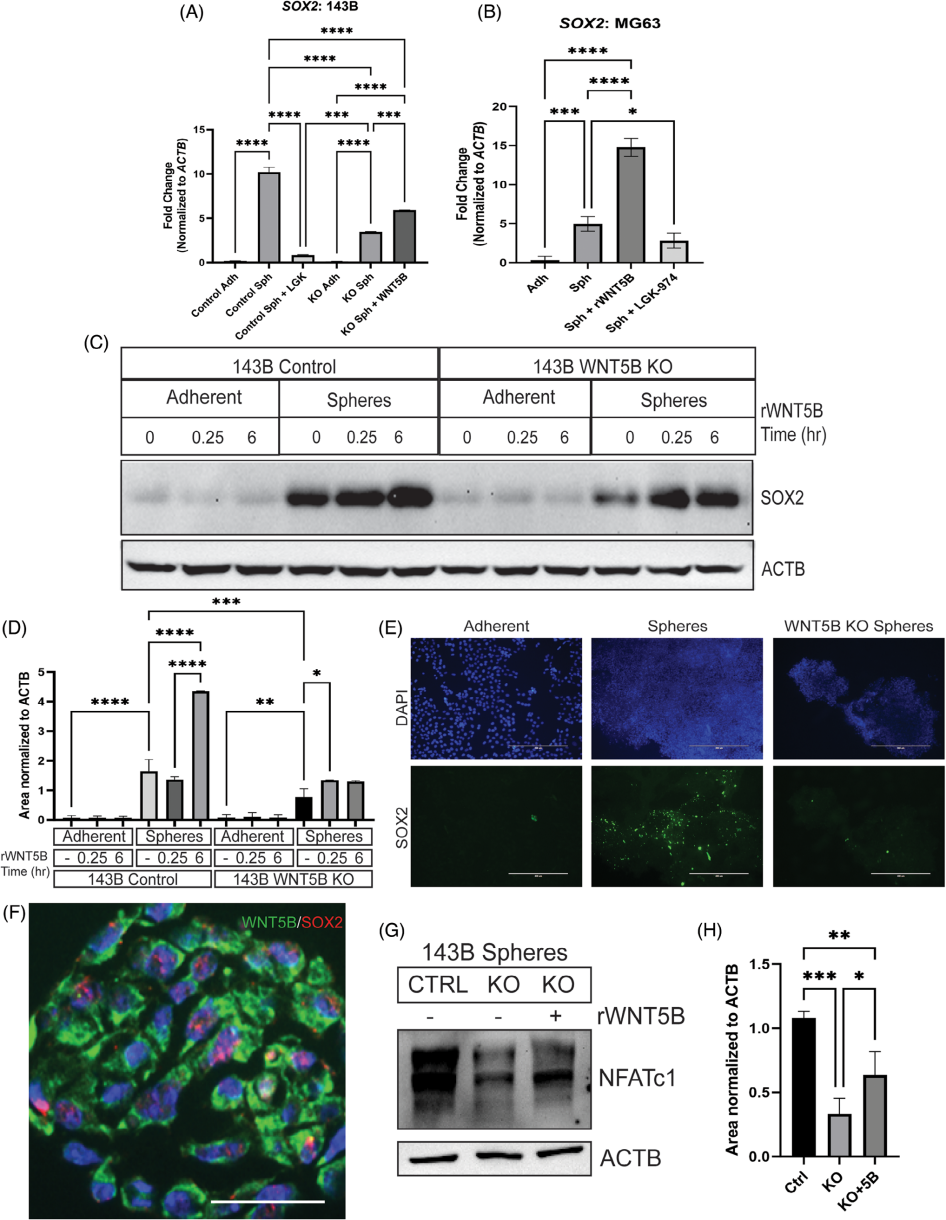
WNT5B induces the expression of SOX2. (A) 143B *WNT5B* KO spheres treated with 10 ng/mL rWNT5B for 6 h compared to untreated 143B *WNT5B* KO and 143B control spheres, Adh = adherent, Sph = sphere, qPCR normalised to *ACTB*, *n* = 3, ***p < .001, ****p < .0001. (B) MG63 spheres treated with 10 ng/mL rWNT5B or 20 µM LGK‐974 for 6 h, Adh = adherent, Sph = sphere, qPCR normalised to *ACTB*, *n* = 3, *p < .05, ***p < .001, ****p < .0001. (C) Western blot of the SOX2 expression in spheres and adherent 143B cells comparing control and *WNT5B* KO cells treated with 50 ng/mL rWNT5B for 15 min or 6 h. Normalised to β‐actin (ACTB), *n* = 3 experimental replicates. (D) Quantification of western blot from (C). *p < .05, **p < .01, ***p < .001, ****p < .0001. (E) Immunofluorescence of 143B adherent cells, 143B spheres, and 143B *WNT5B* KO spheres. 10× microscopy images, SOX2 = green, DAPI nuclear stain = blue. Scale bar = 400 µm. (F) Immunofluorescence of paraffin‐embedded 143B spheres. 40× images, WNT5B = green, SOX2 = red, DAPI nuclear stain = blue. Scale bar = 50 µm. (G) Western blots of NFATc1 and ACTB in 143B spheres comparing control, *WNT5B* KO, and *WNT5B* KO cells treated with 50 ng/mL rWNT5B for 15 min. (H) Quantification of western blots from (G). Normalised to β‐actin (ACTB), *n* = 3 experimental replicates. *p < .05, **p < .01, ***p < .001. KO, Knockout.

*SOX2* expression was significantly reduced in 143B *WNT5B* KO spheres and 143B spheres treated with LGK‐974 compared to that in 143B control spheres (Figure 4A). Treatment with rWNT5B for 6 h was sufficient to partially rescue *SOX2* expression in 143B *WNT5B* KO cells. Likewise, *SOX2* expression in MG63 spheres was significantly enhanced in the sphere population compared to the adherent population, and treatment with rWNT5B increased *SOX2* expression, while inhibition of WNT ligand secretion with LGK‐974 significantly reduced *SOX2* expression (Figure 4B). The relationship of WNT5B with SOX2 was further examined through western blot analysis. Adherent and sphere 143B and 143B *WNT5B* KO cells were treated with 50 ng/mL rWNT5B for 15 min or 6 h and collected. The expression of SOX2 was absent in adherent cells but abundant in CSCs. Additionally, *WNT5B* KO spheres had reduced SOX2 expression, and the protein levels could be rescued with rWNT5B treatment (Figure 4C,D). This result was validated by immunofluorescence, and SOX2 expression was increased in 143B CSCs compared to 143B adherent CSCs and reduced in 143B *WNT5B* KO CSCs (Figure 4E). Additionally, co‐staining of WNT5B and SOX2 in 143B spheres that were embedded in Histogel and sectioned indicated that both WNT5B and SOX2 are expressed in the same cells. WNT5B localises to the cell membrane and cytoplasm, while SOX2 is in the nucleus (Figure 4F).

To elucidate the mechanism of WNT5B regulation of *SOX2*, we analysed the expression of downstream targets of WNT5B.^25^ In pancreatic CSCs, NFATc1 was shown to upregulate *SOX2* expression and then combined with SOX2 as a binding partner to regulate the transcription of epithelial to mesenchymal transition markers and stemness genes.^26^ KO of *WNT5B* led to the reduced protein levels of NFATc1 and this could be rescued by the addition of rWNT5B (Figure 4G,H). Combined, this suggests that NFATc1 may be mediating WNT5B regulation of *SOX2*.

### 3.6 WNT5B enhances chemoresistance to methotrexate

**FIGURE 5:**
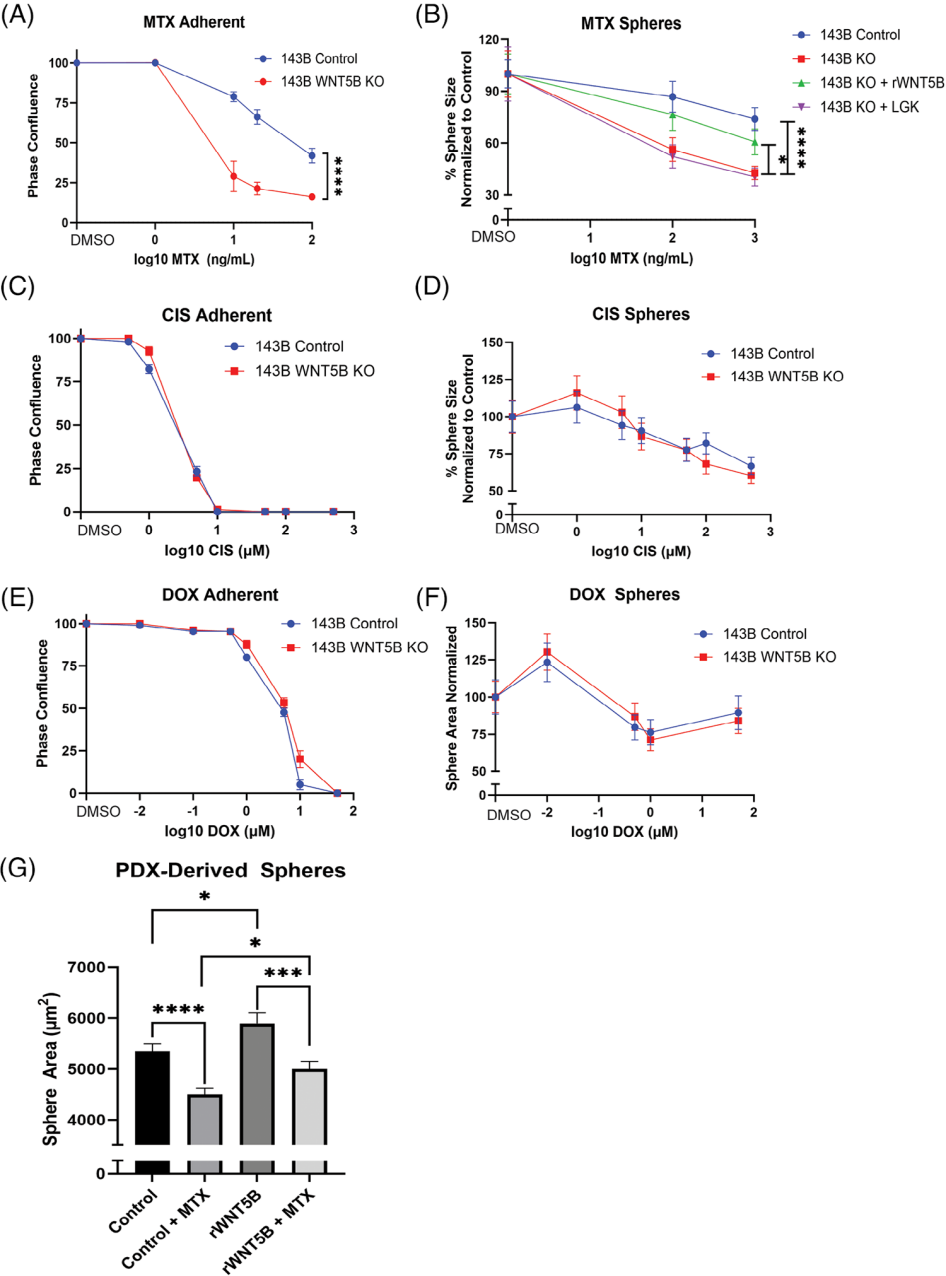
WNT5B regulates osteosarcoma chemoresistance to MTX. (A) Drug response curve of increasing doses of MTX on adherent 143B control and *WNT5B* KO cells. *n* = 3. ****p < .0001. (B) 143B control and *WNT5B* KO spheres treated with vehicle control, 10 ng/mL rWNT5B, 20 µM LGK‐974, and/or MTX at increasing doses. *n* = > 43 spheres per group. *p < .05, ****p < .0001. (C) Adherent 143B control and *WNT5B* KO cells treated with vehicle or CIS at increasing doses. *n* = 3. D) 143B sphere control and *WNT5B* KO cells treated with vehicle or CIS at increasing doses. *n* = > 82 spheres per group. (E) Adherent 143B control and *WNT5B* KO cells treated with vehicle or DOX at increasing doses. *n* = 3. (F) 143B sphere control and *WNT5B* KO cells treated with vehicle or DOX in increasing doses. *n* = > 93 spheres per group. (G) Patient‐derived spheres treated with 50 ng/mL rWNT5B and/or 1000 ng/mL MTX for 72 h. Sphere size quantified with ImageJ. *n* = > 170 spheres per group, *p < .05, ***p < .001, ****p < .0001. MTX, Methotrexate; KO, Knockout.

Since CSCs play a large role in resistance to chemotherapy, we looked at the consequences of knocking out *WNT5B* on susceptibility to MTX, CIS and DOX treatment. In adherent 143B cells, *WNT5B* KO was sufficient to increase sensitivity to MTX by reducing the 143B IC_50_ from 51 to 6 ng/mL (8.5‐fold) (Figure 5A). Likewise, 143B *WNT5B* KO spheres (*WNT5B* KO IC_50_: 306 ng/mL) were more sensitive to MTX than 143B control spheres (control IC_50_: 2641 ng/mL, 8.6‐fold). The addition of rWNT5B to 143B *WNT5B* KO cells (*WNT5B* KO + rWNT5B IC_50_: 1320 ng/mL) significantly increased resistance to MTX, and further inhibition of other WNTs through LGK‐974 (*WNT5B* KO + LGK‐974 IC_50_: 210 ng/mL) did not significantly affect the IC_50_ (Figure 5B). There was no significant change in drug susceptibility to CIS between 143B control and *WNT5B* KO adherent cells or spheres (adherent CIS IC_50_: 2.0 µM control, 2.1 µM *WNT5B* KO; sphere CIS IC_50_: 22.4 µM control, 21.0 µM *WNT5B* KO) (Figure 5C,D). There was also no significant change in drug susceptibility to DOX between 143B control and *WNT5B* KO adherent cells or spheres (adherent DOX Log IC_50_: 0.63 µM control, 0.78 µM *WNT5B* KO; sphere DOX Log IC_50_: 4.6 µM control, 3.7 µM *WNT5B* KO) (Figure 5E,F). Therefore, WNT5B increases resistance in a manner specific to MTX's mechanism of action. Similar to 143B cells, PDX‐derived spheres treated with WNT5B had more resistance to MTX than control spheres not treated with WNT5B (Figure 5G). These results suggest that WNT5B alone is sufficient to increase chemoresistance to MTX.

### 3.7 WNT5B increases metastasis in vivo

**FIGURE 6:**
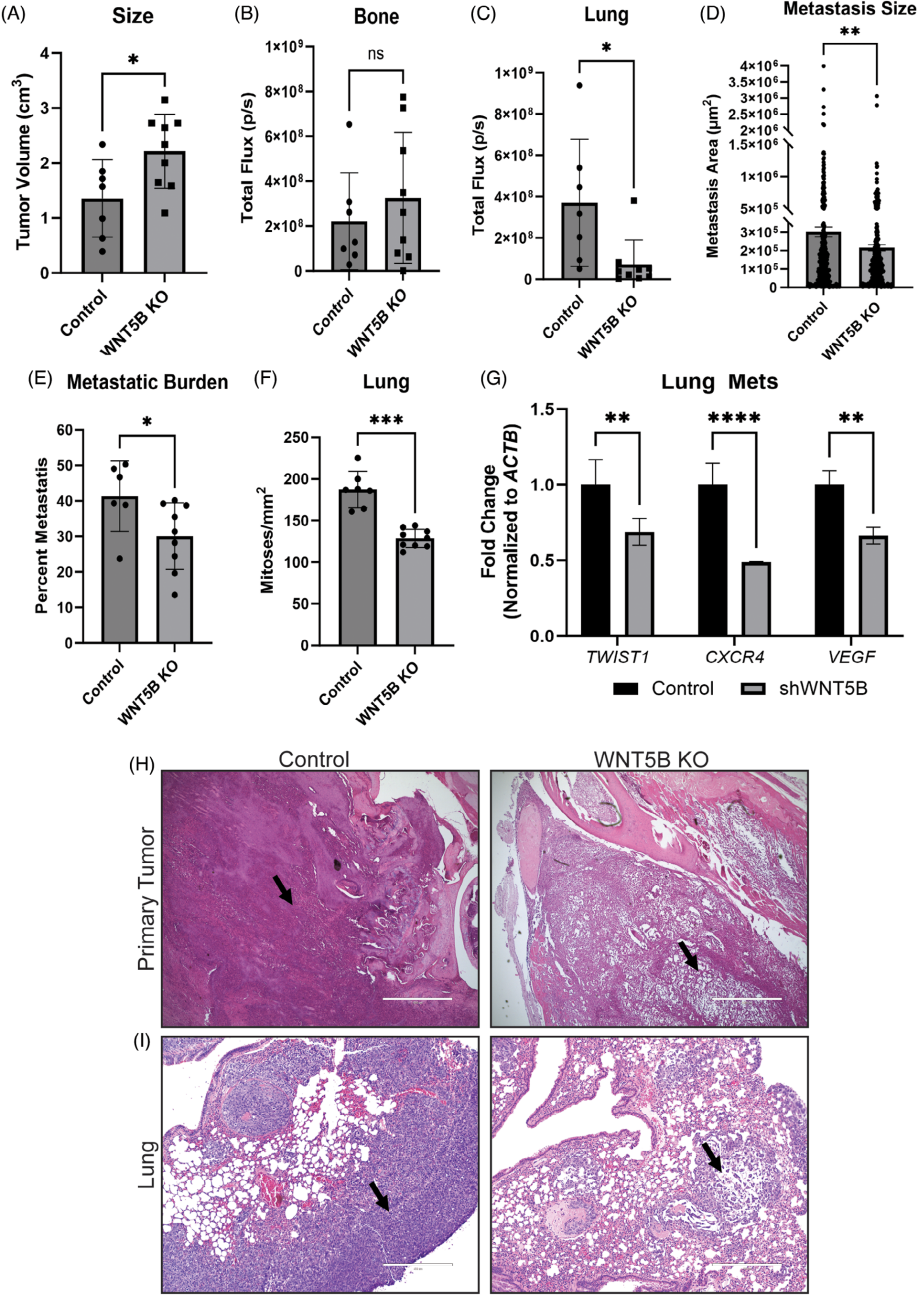
WNT5B enhances lung metastasis and cell density in vivo. (A) 143B control and *WNT5B* KO primary tumours at the last measured volume (cm^3^). *n* = 7 control, *n* = 9 *WNT5B* KO, *p < .05. (B) Ex vivo total flux (photons/second; p/s) measure of luciferase in bones immediately following dissection. *n* = 7 control, *n* = 9 *WNT5B* KO, ns = not significant. (C) Ex vivo total flux measure of lungs immediately following dissection. *n* = 7 control, *n* = 9 *WNT5B* KO, **p < .01. (D) Average area of all metastases from H&E staining of *n* = 7 control, *n* = 9 *WNT5B* KO mice, measured using ImageJ, **p < .01. (E) Percentage of total lung‐bearing metastasis (total metastatic area/total lung area × 100). *n* = 6 control, *n* = 9 *WNT5B* KO, *p < .05. (F) The average count of mitoses/mm^2^ from lung metastases in the control vs. *WNT5B* KO groups. *n* = 7 control, *n* = 9 *WNT5B* KO mice, ***p < .001. (G) qPCR analysis of osteosarcoma lung metastases from mice intratibially injected with 143B control or *WNT5B* knockdown (shWNT5B) cells. Primers are specific to human *TWIST1, CXCR4*, and *VEGF*. The qPCR was normalised to *ACTB, n* = 3. **p < .01, ****p < .0001. (H) 4× H&E staining images of primary bone tumours depicting cell density differences in the control vs. *WNT5B* KO groups. The arrow indicates an area of most visible density differences. Scale bar = 1000 µm. (I) 10× H&E staining images of metastatic lung tumours depicting metastatic size, total burden and cell density differences in the control vs. *WNT5B* KO groups. The arrow indicates an area of most visible density differences. Scale bar = 400 µm. KO, Knockout.

To determine the role of WNT5B on tumour growth in vivo, 143B control and *WNT5B* KO spheres (tagged with luciferase) were dissociated as single cells (5,000 cells), seeded into collagen implants and orthotopically implanted into the tibia of NSG mice using the method developed by Talbot et al.^15^ Tumour growth was measured weekly by IVIS imaging. Primary tumours were slightly larger (1.6× larger at endpoint) in the *WNT5B* KO group compared to the control group (Figure 6A and Figure S4A) but had no significant difference in total flux (photons/second; p/s) as measured by ex vivo luciferase imaging (Figure 6B). In contrast, control tumours metastasised much more than the *WNT5B KO* group (Figure S4B). Additionally, the *WNT5B* KO groups had less photon flux ex vivo than the control group in both the lungs and livers (Figure 6C and Figure S4C). Further, 143B control lungs had a higher percentage of metastatic burden (area of metastasis/total lung area) than *WNT5B* KO lungs, and those metastatic lesions were larger on average in the control mice than in the *WNT5B* KO mice (Figure 6D,E). Control liver and lung metastatic OS tumour deposits were remarkable for significantly more cellularity and mitotic activity when compared with *WNT5B* KO metastases (Figure 6F and Figure S4D). We also tested markers of metastasis: *TWIST1, CXCR4*, and *VEGF*.^27^ All three markers were significantly upregulated in the lungs of mice intratibially injected with 143B control cells compared to mice injected with 143B *WNT5B* knockdown cells (Figure 6G). Hematoxylin and eosin‐stained histologic sections of both control and KO primary bone OS were comprised predominantly of small cells with associated osteoid deposition. Nuclei were round to oval with fine chromatin and prominent nucleoli (Figure 6H). The primary bone tumour was best characterised as the small cell histologic variant of osteosarcoma (SCOS). Interestingly, *WNT5B* KO tumours (primary and metastatic) had markedly less cell density than the control tumours (Figure 6H,I, Figure S4E), which likely explains the difference in photon flux. qPCR analysis of *WNT5B* in metastatic‐burdened lungs post intratibial injection of 143B OS cells showed that the metastases had higher expression of *WNT5B* compared to the parental cell line (Figure S4F). Furthermore, in metastatic OS lung samples of mice injected intratibially with 143B control or *WNT5B* knockdown cells, *SOX2* was reduced by 50% in the knockdown group, indicating a role of WNT5B in the expression of *SOX2* in vivo (Figure S4G). The smaller tumour volume in control tumours compared to *WNT5B* KO tumours suggests that while WNT5B slightly decreases the primary tumour development, it significantly enhances metastasis, as well as cell density, within tumours. This is indicative of a selective pressure on tumour stem cells to metastasise; and, if the ability to metastasise is limited, such as with a reduction in stemness, the primary tumour will continue to grow larger. Clinically, these could be the tumours that have early and aggressive metastases.

### 3.8 WNT5B inhibits glycosaminoglycan accumulation by regulating hyaluronic acid degradation enzymes

**FIGURE 7:**
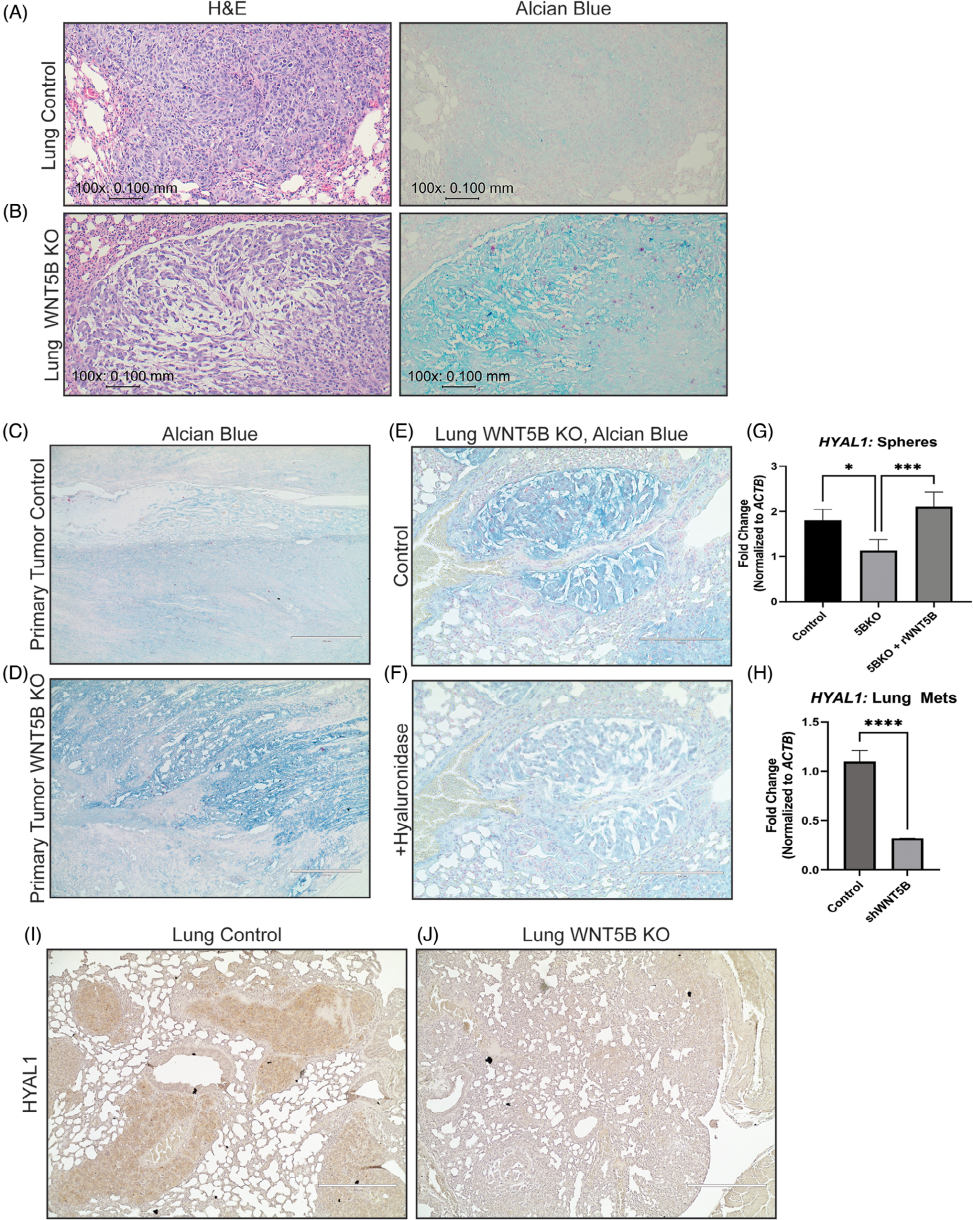
WNT5B inhibits GAG accumulation by increasing the expression of the GAG degradation enzyme HYAL1. (A,B) 100× (10× objective) comparison images of the lung metastases from control and *WNT5B* KO spheres in the same area between H&E and alcian blue staining. Scale bar = .100 mm. (C,D) 10× images of the primary tumours grown from control and *WNT5B* KO spheres stained with alcian blue. Scale bar = 400 µm. (E,F) 20× images of alcian blue stained lung *WNT5B* KO samples incubated with PBS (E) or 1 unit/mL hyaluronidase (F). Scale bar = 200 µm. (G) qPCR of *HYAL1* from 143B control spheres compared to 143B *WNT5B* KO (5BKO) and 143B *WNT5B* KO spheres treated with 50 ng/mL rWNT5B for 6 h. The qPCR was normalised to *ACTB*, *n* = 3, *p < .05, ***p < .001. (H) qPCR analysis of osteosarcoma lung metastases from mice intratibially injected with 143B control or *WNT5B* knockdown (*shWNT5B*) adherent cells, primers specific to human *HYAL1*. The qPCR was normalised to *ACTB, n* = 3 control, *n* = 2 sh*WNT5B*. ****p < .0001. (I,J) 10× images of lung metastases from control and *WNT5B* KO spheres stained with an antibody to HYAL1. Scale bar = 400 µm. GAG, Glycosaminoglycans; KO, knockout.

The striking difference in cellularity and spacing of the *WNT5B* KO tumours led us to hypothesise that glycosaminoglycans (GAGs) could be involved. GAGs are negatively charged polysaccharides that are highly implicated as regulators of the tumour microenvironment.^28^ Triantafyllou et al. described the histology of pleomorphic salivary gland adenomas, and the GAGs and cellular morphology seen in that review mimicked the phenotype in our OS samples.^29^ Therefore, lungs, primary bone tumours and livers were stained with alcian blue (pH 2.5) to assess GAG deposition (Figure 7A–D and Figure S4H,I). *WNT5B* KO metastases and primary samples demonstrated increased alcian blue‐positive GAG‐like material in the extracellular matrix (Figure 7A–D and Figure S4H,I). Hyaluronic acid, a GAG, has been implicated as a regulator of tumour metastasis.^30^ Therefore, to determine the specific GAG, we incubated the tumours with hyaluronidase, before staining them with alcian blue (Figure 7E,F). There was a significant decrease in alcian blue staining in the presence of hyaluronidase, indicating the presence of hyaluronic acid. Then, qPCR was performed to detect hyaluronic acid regulatory genes. Interestingly, the GAG degradation enzyme hyaluronidase 1 (*HYAL1*) was significantly upregulated in 143B control spheres compared to *WNT5B* KO spheres, and the addition of 50 ng/mL rWNT5B was sufficient to rescue this expression (Figure 7G). In agreement with our in vitro sphere gene expression data, in metastatic OS lung samples of mice injected intratibially with 143B control or *WNT5B* knockdown cells, *HYAL1* was significantly decreased in the *WNT5B* knockdown cells (Figure 7H). Additionally, *WNT5B* KO metastases demonstrated less expression of HYAL1 by IHC compared to control metastases (Figure 7I,J). These results suggest that WNT5B regulates hyaluronic acid levels, possibly contributing to increased metastasis.^30^

### 3.9 ROR1 is a therapeutic target for WNT5B inhibition in osteosarcoma stem cells

**FIGURE 8:**
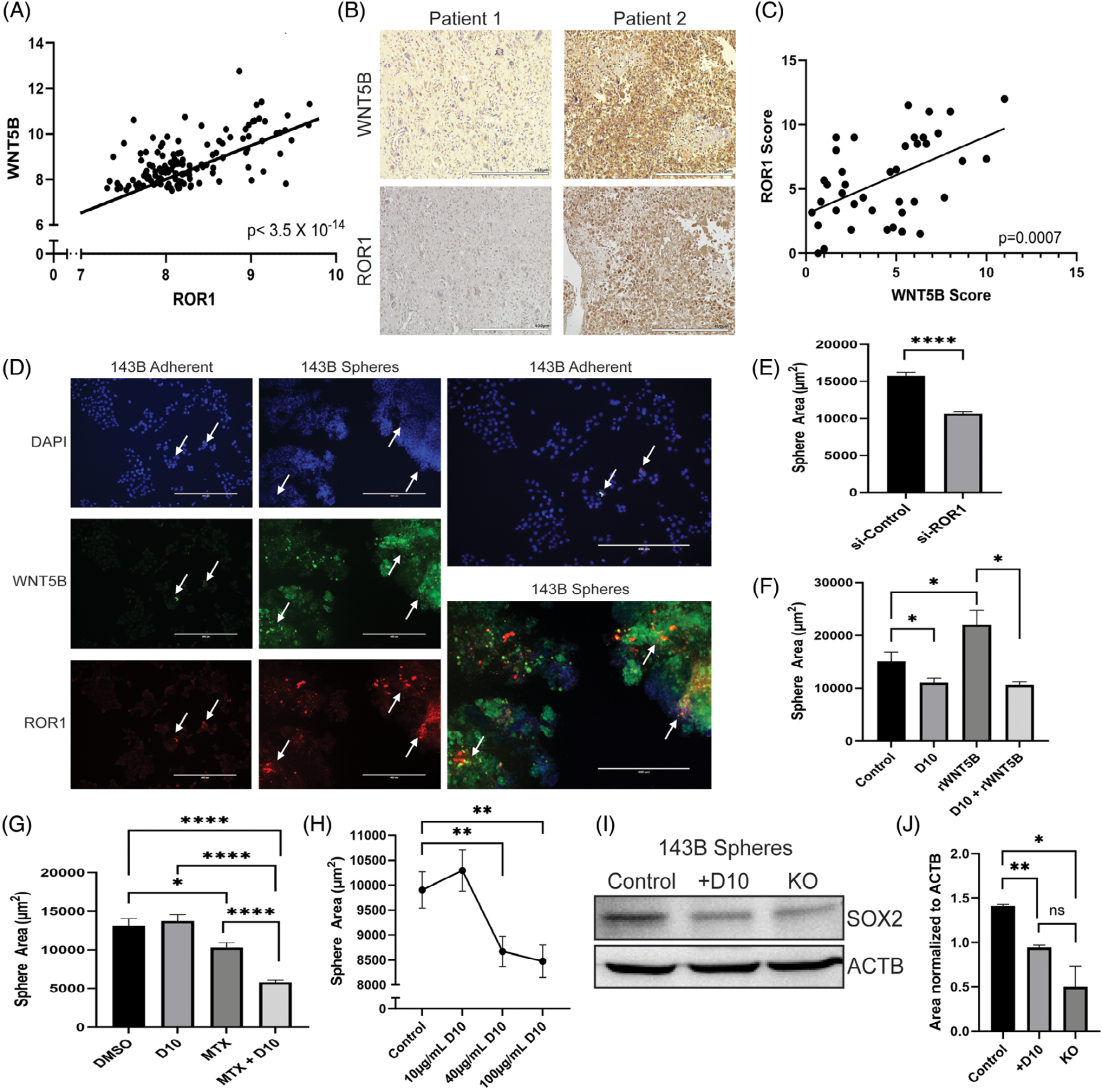
ROR1 is a therapeutic target for osteosarcoma stem cells. (A) Expression array correlation data showing a significantly positive correlation between *WNT5B* and *ROR1* in osteosarcoma samples. Data were analysed using the R2 genomics analysis and visualization platform, and data generated by Kuijjer et al.^18^ *n* = 127, *p* < 3.5 × 10^−14^. (B) Representative images of immunohistochemistry on an osteosarcoma tissue microarray stained for ROR1 and WNT5B. Matched images from two samples, one low for both WNT5B and ROR1 and one high for both WNT5B and ROR1. *n* = 40 cores in duplicate, scale bar = 400 µm. (C) Correlation graph of osteosarcoma tissue microarray depicted in (B. Average immune reactive scoring of all WNT5B and ROR1 cores indicates a positive correlation between WNT5B and ROR1 in osteosarcoma patient samples. *n* = 40 cores, *p* = .0007. (D) Immunofluorescence of 143B adherent cells and spheres. 10× microscopy images, WNT5B = green, ROR1 = red, DAPI nuclear stain = blue. White arrows indicate areas of WNT5B/ROR1 colocalisation (yellow), scale bar = 400 µm. (E) 143B spheres treated with siRNA to *ROR1* or scrambled siRNA for 72 h. Sphere size quantified using ImageJ. *n* = > 380 spheres per group, ****p < .0001. (F) 143B spheres treated with 100 µg/mL D10 and/or 50 ng/mL rWNT5B for 48 h. The sphere area was quantified using ImageJ. *n* ≥ 80 spheres per group, *p < .05. (G) 143B spheres treated with a low dose of (40 µg/mL) D10 and/or high dose (10 µg/mL) MTX for 48 h to assess reduction in chemoresistance. The sphere area was quantified using ImageJ. *n* ≥ 110 spheres per group, *p < .05, ****p < .0001. (H) PDX‐derived spheres treated with increasing doses (0, 10, 40, 100 µg/mL) of D10. Sphere area quantified using ImageJ. *n* ≥ 210 spheres per group, **p < .01. (I) SOX2 western blot of 143B spheres control, spheres treated with 40 µg/mL D10 for 48 h, and 143B *WNT5B* KO spheres. (J) ImageJ quantification of the western blot in (I). *p < .05, **p < .01, ns = not significant. Normalised to β‐Actin, *n* = 3 experimental replicates. PDX, Patient‐derived xenograft; MTX, methotrexate.

Since these data have shown an important role for WNT5B in promoting the OS stem cell subpopulation, as well as metastasis, we sought to find a receptor to target its signalling. Using publicly available data,^18^, ^31^ we found that the expression of *ROR1*, one of the noncanonical WNT receptors, positively correlates with the expression of *WNT5B* in 127 patients with OS (Figure 8A). In contrast, neither *ROR2* nor *RYK* receptors correlate with *WNT5B* expression (Figure S5A,B). IHC was performed on ROR1 with the same tissue microarray used in Figure 1 and showed a strong positive correlation between WNT5B and ROR1 in OS patient samples (Figure 8B,C). Furthermore, ROR1 is strongly expressed in the OS cell lines MG63, SAOS‐2 and 143B. In contrast, ROR1 was not expressed in normal human osteoblasts (Figure S5C,D). In addition, ROR1 was upregulated in the 143B spheres and colocalised with WNT5B, as shown by immunofluorescence (Figure 8D). siRNA knockdown of *ROR1* showed that, similar to WNT5B, *ROR1* knockdown significantly reduced sphere size (Figure 8E). Therefore, we hypothesised that ROR1 is a viable and cancer‐specific target and began to target WNT5B through ROR1 inhibition.

D10 is an inhibitory monoclonal antibody to ROR1.^32^ 100 µg/mL D10 significantly reduced the size of 143B and MG63 spheres and overcame the addition of rWNT5B to continue reducing sphere size (Figure 8F and Figure S5E). Next, the effect of D10 on rWNT5B‐induced chemoresistance was examined. With a low dose (40 µg/mL) of D10, the 143B sphere size was not changed; however, in combination with 10 µg/mL MTX, D10 reduced the sphere size significantly more than MTX alone, indicating a reduction in chemoresistance (Figure 8G). Furthermore, PDX‐derived spheres also decreased in size in response to 40 µg/mL D10 treatment, which are more sensitive than established OS cell lines (Figure 8H). Finally, inhibition of WNT5B signalling through ROR1 was tested to determine its effects on SOX2 in OS spheres. 143B spheres were treated with 40 µg/mL D10 for 48 h and immunoblotted to detect SOX2. SOX2 was markedly decreased in the D10‐treated group, almost to the level of the 143B *WNT5B* KO spheres (Figure 8I,J). Altogether, these results suggest that inhibition of WNT5B through ROR1 should be further explored as a potential addition to the current therapeutic regimen.

## 4 DISCUSSION

Here, we identified WNT5B as the most upregulated WNT in OS and described its role in the CSC subpopulation. WNT5B enhances OS sphere size, migration, and sphere‐forming efficiency. Additionally, this study shows that WNT5B drives drug resistance to MTX in vitro *and* enhances metastasis and cellular density in vivo.

The CSC hypothesis is a theory that suggests that within a tumour, a small population of stem‐like cells exists that is largely responsible for tumour initiation, maintenance, metastasis, and chemoresistance.^33^ OS sarcospheres have been described since 2005 and have been shown to be tumourigenic in in vivo models that increase chemoresistance and mimic the CSC phenotype.^23^ This makes sarcospheres useful and relevant tools for understanding the CSC population in OS.^6^ However, there are still controversies in the field of CSC assays,^34^ which is a limitation of this study. Several widely accepted stemness genes have been reported to play important roles in the CSC population, including *SOX2*, *OCT3/4*, and *NANOG*.^6^ Of note, in paediatric sarcomas, SOX2 was found to be the most associated with tumour initiation and growth, making cells with high expression of SOX2 the most fitting of the CSC phenotype.^35^ SOX2 has also been implicated as essential for OS tumour growth and cell proliferation.^36^ This is the first study to demonstrate that WNT5B regulates *SOX2* expression in CSCs.

WNT5B has been implicated in the CSC population in a few other cancers, even though its role in OS stem cells had not been explored.^10^ We add to the current knowledge of WNT5B in CSCs to show that WNT5B drives OS stemness through *SOX2* modulation and that KO of *WNT5B* can inhibit stem cells through reduction of sphere size, migration, sphere‐forming efficiency, and MTX resistance. WNT5B enhancing resistance to MTX, and not DOX or CIS, implies a WNT5B/MTX‐specific mechanism of resistance, which warrants further study into the associated pathway.

The major cause of death for OS patients is pulmonary metastasis^3^; therefore, understanding the molecular drivers of lung metastasis is essential. Only a few papers discuss the role of WNT5B in metastasis for other cancers.^10^ A study in oral squamous cell carcinoma described that patients with lymph node metastases had higher mRNA and protein expression of WNT5B.^37^ Our work has shown for the first time that WNT5B drives OS stem cell metastatic capability and that through KO of *WNT5B*, metastasis and mitotic activity are decreased. Interestingly, the primary tumour growth rate was slightly lower in the control group than in the *WNT5B* KO group. While this finding was initially unexpected, the lack of phenotypic differences in adherent cells indicated that stemness is the primary target of WNT5B in OS. Therefore, the primary tumour results were not as imperative to the study at hand, and the difference in metastasis was the most striking. Additionally, *WNT5B* KO did not completely abolish metastasis. Our model system KO *WNT5B* from tumour cells and not the host environment. Thus, it is possible that paracrine WNT5B signalling from the immune system or tumour microenvironment could contribute to metastatic growth, as evidenced in ovarian CSCs.^38^

Our in vitro and in vivo models demonstrate an increase in GAG accumulation, specifically hyaluronic acid, in the *WNT5B* KO group and an increase in the degradation enzyme *HYAL1* (hyaluronidase 1) in the control group, correlating with increased metastasis. While the regulation of GAGs is very complex and finely regulated, especially in cancer, some literature suggests that HYAL1 is essential for metastasis and tumour growth.^30^ In contrast, high molecular weight hyaluronic acid, such as found in naked mole rats that are resistant to cancer, has tumour‐suppressive properties.^39^

One of the receptor tyrosine kinases through which WNT5B can signal,^25^ ROR1, has gained increasing attention in the field of therapeutic targets. ROR1 has been implicated in cancers such as leukaemia, breast cancer and ovarian cancer as a regulator of tumour cell survival, drug resistance, stemness, and proliferation.^40^ One feature of ROR1 that makes it such an enticing target is its absence in normal postnatal tissue.^41^ Zilovertamab (formerly known as cirmtuzumab or UC‐961) is a monoclonal antibody to ROR1 and is in clinical trials for the treatment of leukaemias and other cancers, such as prostate cancer and lung cancer.^42^, ^43^ D10 has been studied for its efficacy in inhibiting WNT5A in breast cancer and leukaemia but never against WNT5B signalling or in OS.^32^, ^44^

Our analysis of publicly available data revealed that *ROR1* (not *RYK* or *ROR2*) was the only receptor to positively correlate with *WNT5B* in OS patient samples. Here, we show that inhibition of ROR1 with D10 treatment in OS stem cells, similar to *WNT5B* KO, reduces sphere size, MTX resistance and SOX2 expression.

The morphologic difference in vivo between control and *WNT5B* KO cell density warrants further study into the mechanistic implications of WNT5B inhibition. It is possible that WNT5B enhances the CSC's ability to communicate with neighbouring healthy tissue to continue to feed off growth factors in the environment, thereby increasing cell survival and cell density. Particularly interesting is the drastic difference in lung metastasis, both morphologically and quantitatively. This suggests a role for WNT5B in metastatic seeding and/or proliferation, especially via stem cells. Together, this study highlights WNT5B as a candidate for therapeutically targeting OS stem cells, thereby preventing drug resistance and metastasis.

## AUTHOR CONTRIBUTIONS

Rachel S. Perkins and Susan A. Krum designed, executed, and managed the project. Rachel S. Perkins, Glenn Murray, Sarocha Suthon, Lindsey Davis, Nicholson B. Perkins III, Lily Fletcher, Amanda Bozzi, Saylor L. Schreiber, Jianjian Lin, Rahul R. Pillai, and Alec J. Wright performed and analysed experiments. Rachel S. Perkins prepared the figures for the manuscript. Rachel S. Perkins and Susan A. Krum wrote the manuscript. Rachel S. Perkins, Gustavo A. Miranda‐Carboni, and Susan A. Krum edited the manuscript. All authors reviewed and approved the final manuscript.

## CONFLICT OF INTEREST STATEMENT

The authors declare no conflicts of interest.

## ETHICAL APPROVAL

All animal studies were approved by UTHSC's Institutional Animal Care and Use Committee.

## Supporting information

Supporting Information

## Data Availability

No sequencing data were generated as part of this paper. All uses of publicly available data are referenced in Section 2.7.
