# Supplementary material for: WNT5B drives osteosarcoma stemness, chemoresistance and metastasis
Source: Clin Transl Med. 2024 Apr 30;14(5):e1670. doi: 10.1002/ctm2.1670 (PMC11061378; doi:10.1002/ctm2.1670)
Supplement: Supplementary file 1 — Supporting Information [file CTM2-14-e1670-s001.docx]

***
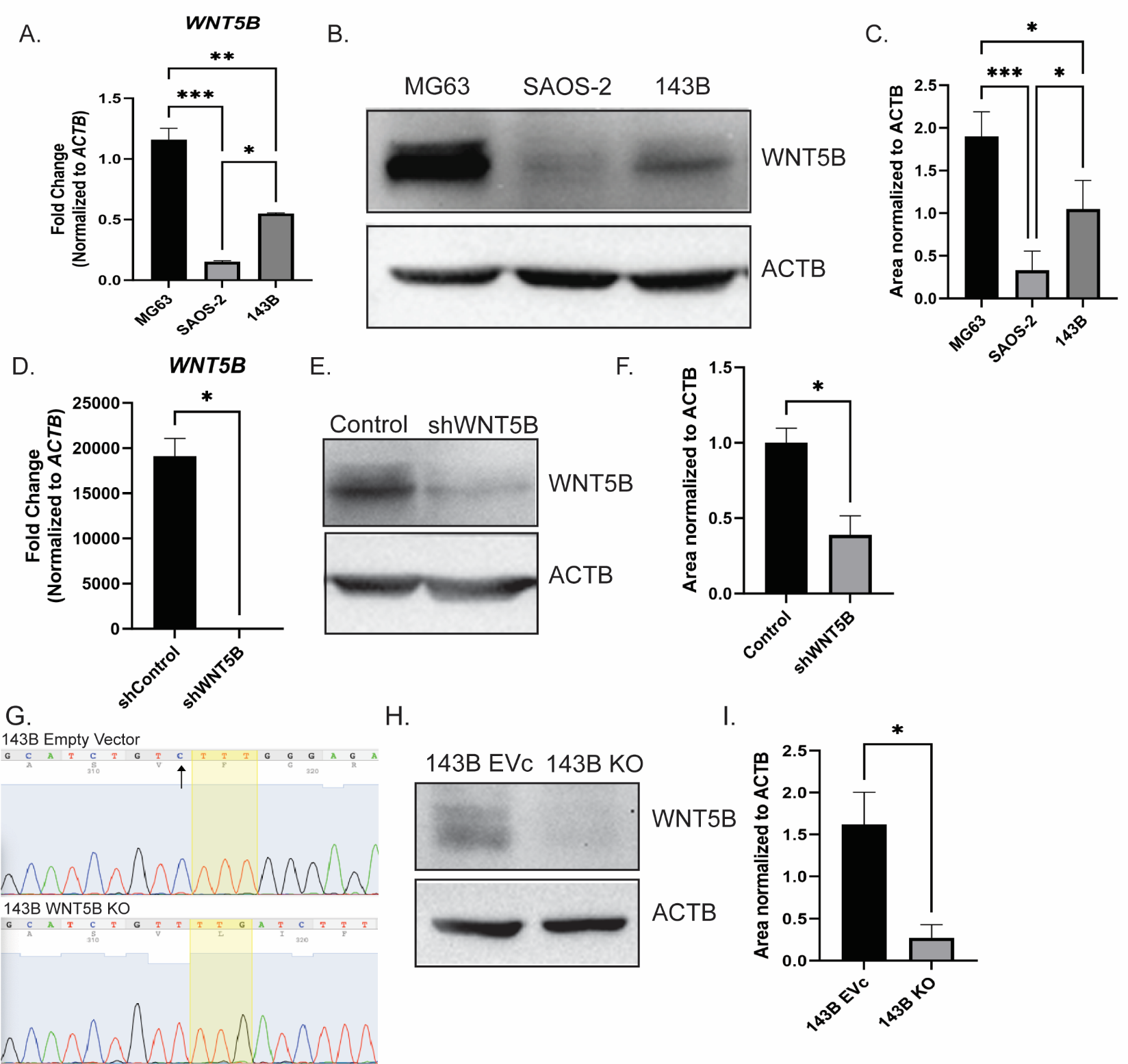
***

***Supplemental Figure 1: WNT5B is expressed at varying levels across osteosarcoma cell lines and can be successfully knocked out in 143B cells***

A) RT‒qPCR to quantitate *WNT5B* expression in human osteosarcoma cell lines. The qPCR was normalized to *ACTB,* n=3, **p<0.01 and ***p<0.001. B) Western blot using an antibody against WNT5B in human osteosarcoma cell lines. Normalized to β-actin (ACTB), n=3 experimental replicates. C) ImageJ quantification of the western blots in part B. *p<0.05, ***p<0.001. D) RT‒qPCR to quantitate *WNT5B* after 143B short hairpin (sh) knockdowns of *WNT5B* compared to a sh-scrambled control. The qPCR was normalized to *ACTB*, n=3, *p<0.05. E) Western blot using an antibody to WNT5B after sh-*WNT5B* knockdown compared to sh-control, n=3. F) ImageJ quantification of the western blots in part D. *p<0.05. G) DNA sequencing peaks of 143B CRISPR Empty Vector Control (Top) and CRISPR *WNT5B* KO (Bottom) showing deletion of “C” leading to frameshift mutation from F to L. H) Western blot using an antibody to WNT5B after transfection of the CRISPR Empty Vector Control (143B EVc) or CRISPR *WNT5B* KO (143B KO). Normalized to β-actin (ACTB), n=3 experimental replicates. I) ImageJ quantification of the western blot in part H, n=3 experimental replicates, *p<0.05.


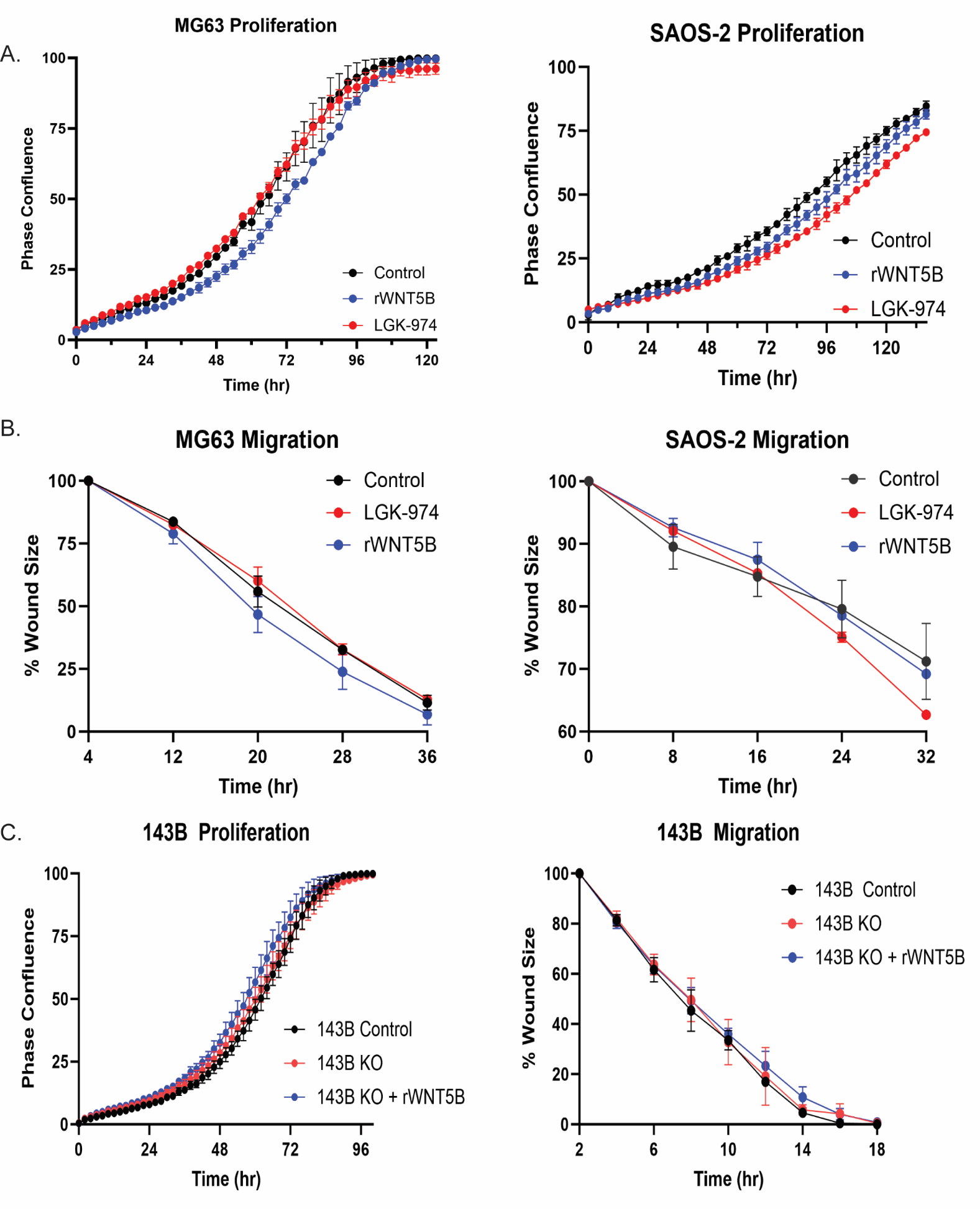


***Supplemental Figure 2: WNT5B does not affect adherent osteosarcoma cell proliferation or migration***

A) Proliferation rate of MG63 and SAOS-2 adherent cell lines treated with either 10 ng/mL rWNT5B or 10 μM LGK-974. n=3 wells/group. B) Wound healing of MG63 and SAOS-2 adherent cell lines treated with either 10 ng/mL rWNT5B or 10 μM LGK-974. n=3 wells/group C) Proliferation and wound healing rates of 143B Control and *WNT5B* KO mice treated with 10 ng/mL rWNT5B, n=3 wells/group.


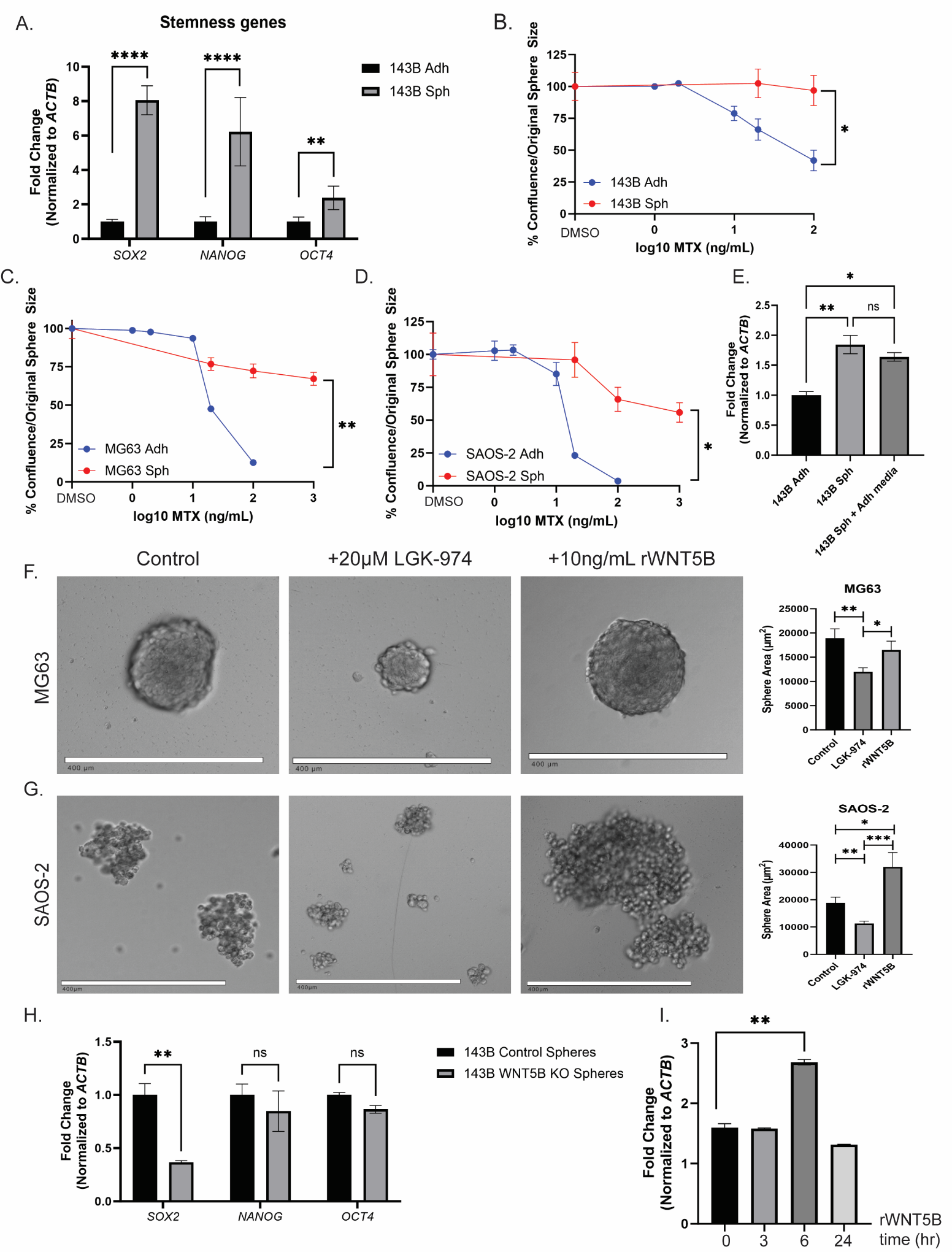


***Supplemental Figure 3: WNT5B induces stemness in osteosarcoma spheres through enhancement of sphere size and the stemness gene SOX2***

A) 143B spheres vs. adherent cells’ expression of the stemness genes *SOX2, NANOG,* and *OCT4*. qPCR data were normalized to *ACTB,* and adherent samples were normalized to 1, Adh = adherent, Sph = Sphere. n=3, **p<0.01, ****p<0.0001. B) 143B adherent cells and spheres treated with vehicle control and MTX at increasing doses for 72 hours. n=3. Adherent cells were plotted as percentage confluence normalized to the DMSO control. Spheres were plotted as the percentage sphere area of the DMSO control, *p<0.05. C) MG63 adherent cells and spheres treated with vehicle control or MTX at increasing doses for 72 hours. n=3. Adherent cells were plotted as percentage confluence normalized to the DMSO control. Spheres were plotted as the percentage sphere area of the DMSO control, **p<0.01. D) SAOS-2 adherent cells and spheres treated with vehicle control or MTX at increasing doses for 72 hours. n=3. Adherent cells were plotted as the percentage confluence normalized to the DMSO control. Spheres were plotted as the percentage sphere area of the DMSO control, *p<0.05. E) Adherent cells and spheres were grown with the indicated media*.* qPCR was performed for *WNT5B* and data were normalized to *ACTB,* and adherent samples were normalized to 1, Adh = adherent, Sph = Sphere. *p<0.05, **p<0.01, ns = not significant. F) MG63 spheres treated with either 20 µM LGK-974 or 10 ng/mL rWNT5B for 72 hours. Sphere size quantified using ImageJ. n= >60 spheres per group, *p<0.05, **p<0.01, Scale bar = 400 µm. G) SAOS-2 spheres treated with either 20 µM LGK-974 or 10 ng/mL rWNT5B for 72 hours. Sphere size quantified using ImageJ. n= >45 spheres per group, *p<0.05, **p<0.01, ***p<0.001, Scale bar = 400 µm. H) qPCR analysis of stemness genes in 143B control versus *WNT5B* KO spheres. qPCR normalized to *ACTB*, 143B control spheres normalized to 1, n=2, **p<0.01. I) qPCR analysis of *SOX2* in 143B control spheres treated with 10 ng/mL rWNT5B for 3, 6, or 24 hours. qPCR normalized to *ACTB*, n=3, **p<0.01.


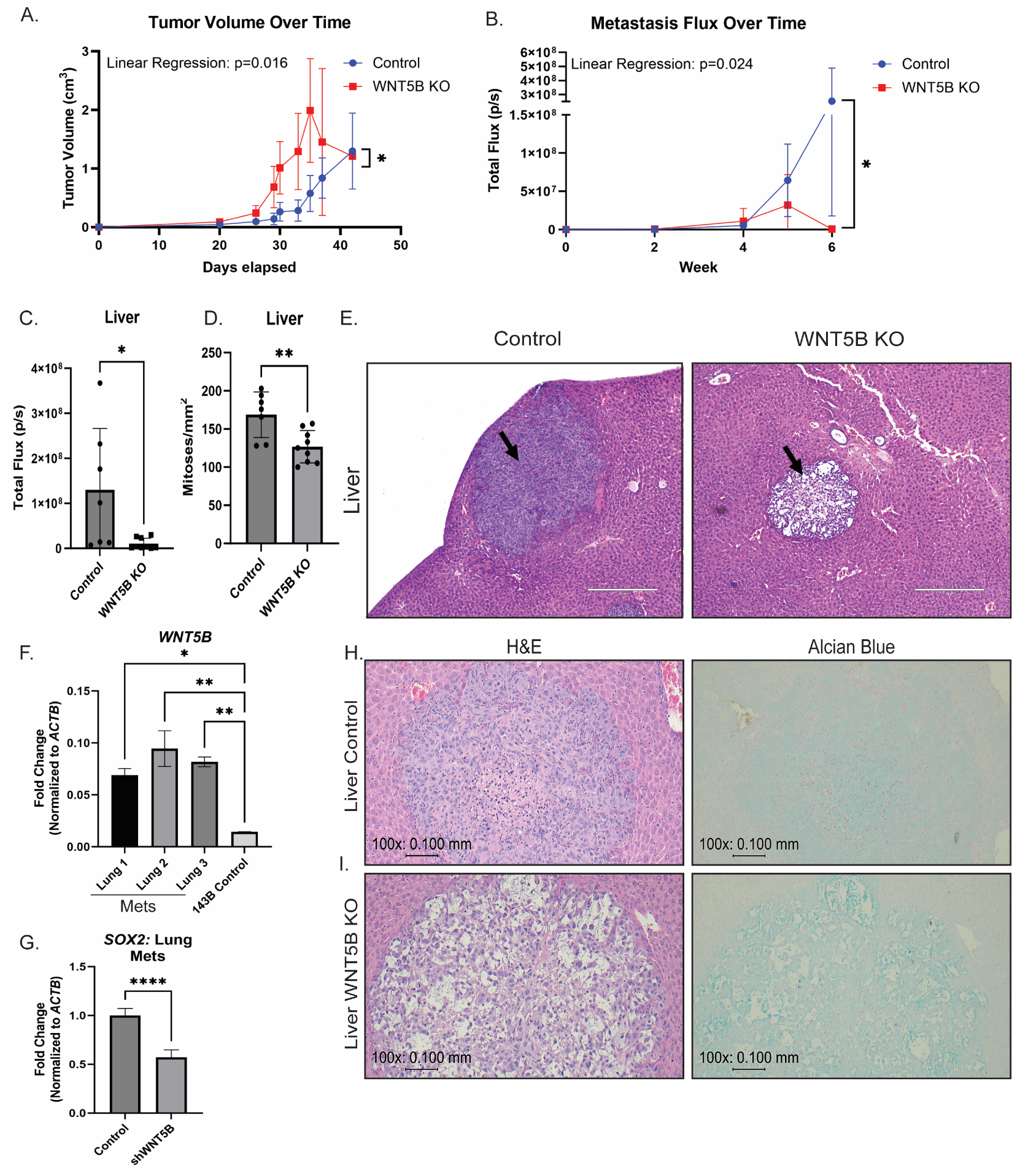


***Supplemental Figure 4: WNT5B enhances liver metastasis and cell density in vivo***

A) Tumor volume over time measured in cm^3^. Simple linear regression: p = 0.016, n=7 control, n=9 *WNT5B* KO mice *p<0.05. B) Metastatic total flux *in vivo* (p/s) measured over time. Simple linear regression: p = 0.024, n=6 control, n=8 *WNT5B* KO mice. *p<0.05. C) *Ex vivo* total flux measure of livers immediately following dissection. n=7 control, n=9 *WNT5B* KO, **p<0.01. D) Mitoses/mm^2^ average count from liver metastases in the control vs. *WNT5B* KO groups. n=7 control, n=9 *WNT5B* KO mice, **p<0.01. E) 10X H&E staining images of metastatic liver tumors depicting cell density differences in the control vs *WNT5B* KO groups. The arrow indicates an area of most visible density differences. Scale bar = 400 µm. F) qPCR analysis of osteosarcoma lung metastases compared to the *in vitro* cells prior to injection. The primers are specific to human *WNT5B*, n=3. *p<0.05, **p<0.01.- G) qPCR analysis of osteosarcoma lung metastases from mice intratibially injected with 143B control or *WNT5B* knockdown (shWNT5B) adherent cells, primers specific to human *SOX2*, n=3 Control, n=2 shWNT5B. ****p<0.0001 H-I) 100X (10X objective) comparison images of liver control/*WNT5B* KO metastases in the same area between H&E and alcian blue staining. Scale bar = 0.100 mm.


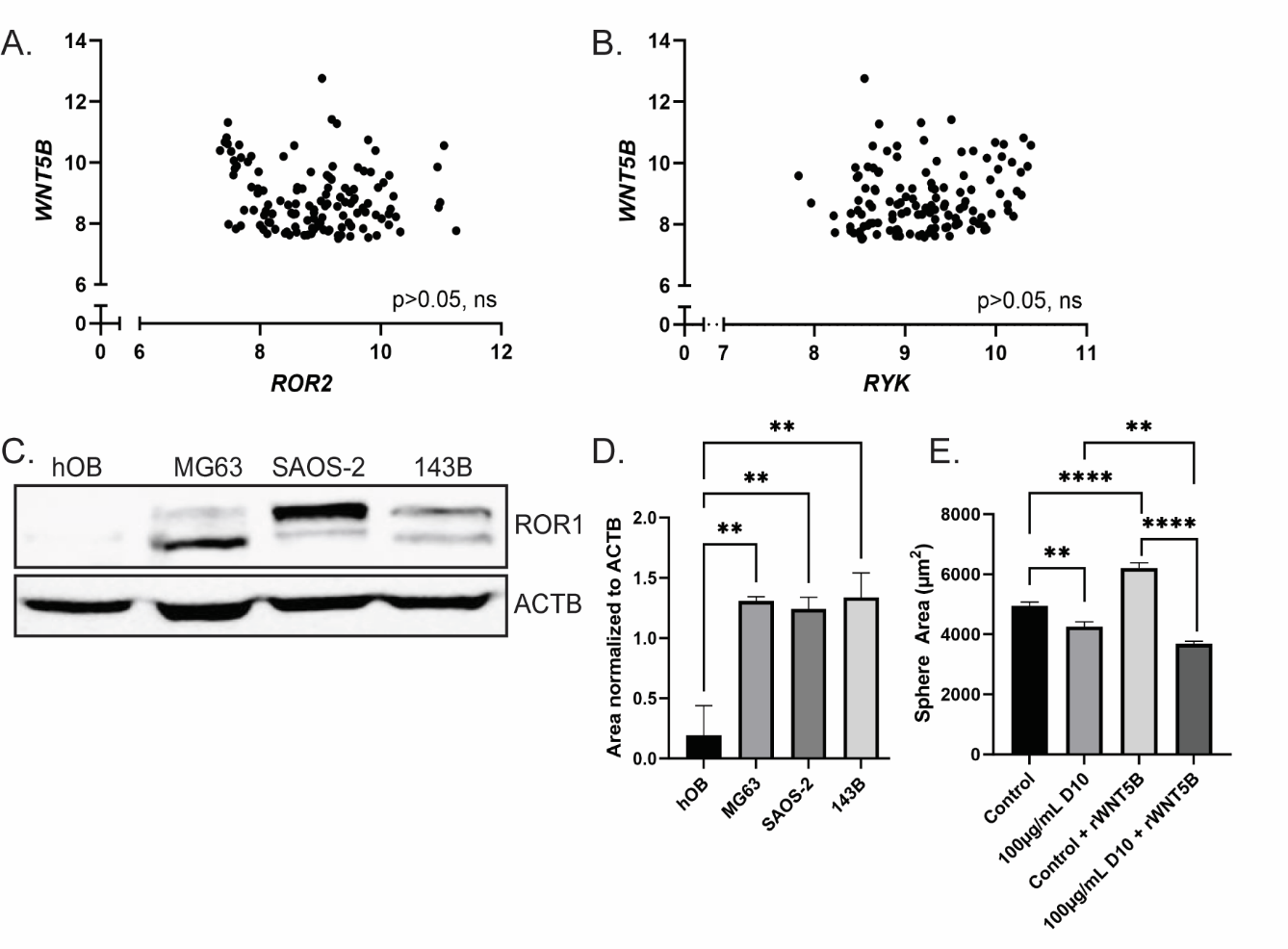


***Supplemental Figure 5: ROR1 is a viable target for inhibiting WNT5B in osteosarcoma***

A) Expression array correlation data showing no correlation between *WNT5B* and *ROR2* in osteosarcoma samples. Data were analyzed using the R2 Genomics Analysis and Visualization Platform from data generated by Kuijjer *et al*.^18^ n=127, ns = not significant B) Expression array correlation data showing no correlation between *WNT5B* and *RYK* in osteosarcoma samples. Data were analyzed using the R2 Genomics Analysis and Visualization Platform from data generated by Kuijjer *et al*.^18^ n=127, ns = not significant. C) Western blot analysis of ROR1 in healthy human osteoblasts (hOBs) and MG63, SAOS-2, and 143B osteosarcoma cell lines. Normalized to β-actin (ACTB), n=3 experimental replicates. D) ImageJ quantification of the western blots in part C. **p<0.01. E) MG63 spheres treated with 100 µg/mL D10 and/or 50 ng/mL rWNT5B for 48 hours. The sphere area was quantified using ImageJ. n= >140 spheres per group, *p<0.05.


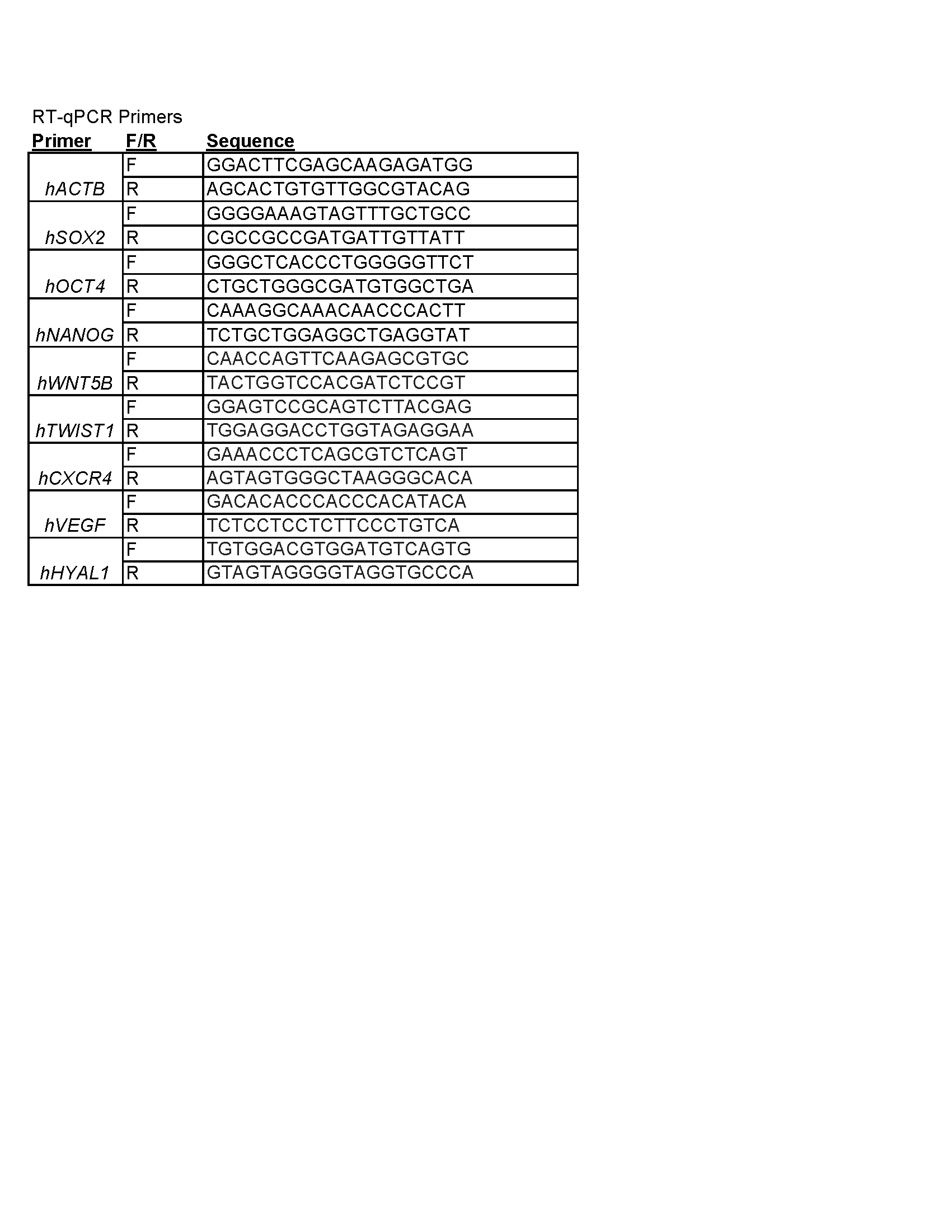


***Supplemental Table 1: Primers***

Abbreviations: h = human, F= forward, R = reverse





***Supplemental Table 2: Antibodies***

Abbreviations: WB = western blot, IF = immunofluorescence, IHC = immunohistochemistry, Ms = mouse, Rb = rabbit, MW = molecular weight, NGS = normal goat serum, BSA = bovine serum albumin
